# Tackling Humidity with Designer Ionic Liquid-Based Gas Sensing Soft Materials

**DOI:** 10.1002/adma.202107205

**Published:** 2022-01-09

**Authors:** Carina Esteves, Susana I. C. J. Palma, Henrique M. A. Costa, Cláudia Alves, Gonçalo M. C. Santos, Efthymia Ramou, Ana Luísa Carvalho, Vitor Alves, Ana C. A. Roque

**Affiliations:** Associate Laboratory i4HB – Institute for Health and Bioeconomy, NOVA School of Science and Technology, NOVA University of Lisbon, Caparica 2829-516, Portugal; UCIBIO – Applied Molecular Biosciences Unit, Department of Chemistry, NOVA School of Science and Technology, NOVA University of Lisbon, Caparica 2829-516, Portugal; LEAF – Linking Landscape, Environment, Agriculture and Food, Instituto Superior de Agronomia, Universidade de Lisboa, Lisboa 1349-017, Portugal; Associate Laboratory i4HB – Institute for Health and Bioeconomy, NOVA School of Science and Technology, NOVA University of Lisbon, Caparica 2829-516, Portugal; UCIBIO – Applied Molecular Biosciences Unit, Department of Chemistry, NOVA School of Science and Technology, NOVA University of Lisbon, Caparica 2829-516, Portugal

**Keywords:** anion-tunability, gas sensing, gelatin, humidity, ionogels, liquid crystals, methylimidazolium ionic liquids

## Abstract

Relative humidity is simultaneously a sensing target and a contaminant in gas and volatile organic compound (VOC) sensing systems, where strategies to control humidity interference are required. An unmet challenge is the creation of gas-sensitive materials where the response to humidity is controlled by the material itself. Here, humidity effects are controlled through the design of gelatin formulations in ionic liquids without and with liquid crystals as electrical and optical sensors, respectively. In this design, the anions [DCA]^−^ and [Cl]^−^ of room temperature ionic liquids from the 1-butyl-3-methylimidazolium family tailor the response to humidity and, subsequently, sensing of VOCs in dry and humid conditions. Due to the combined effect of the materials formulations and sensing mechanisms, changing the anion from [DCA]^−^ to the much more hygroscopic [Cl]^−^, leads to stronger electrical responses and much weaker optical responses to humidity. Thus, either humidity sensors or humidity-tolerant VOC sensors that do not require sample preconditioning or signal processing to correct humidity impact are obtained. With the wide spread of 3D- and 4D-printing and intelligent devices, the monitoring and tuning of humidity in sustainable biobased materials offers excellent opportunities in e-nose sensing arrays and wearable devices compatible with operation at room conditions.

## 1 Introduction

Humidity is ubiquitous, as water is part of all systems on Earth, from soils and air to animals and humans. Monitoring relative humidity (RH) is relevant in a wide variety of fields, including industrial processing and manufacturing, environment and agriculture, and biometric and medical applications.^[1,2]^ For example, RH fluctuations in exhaled air during breathing or speech can be a source of information on respiratory diseases (e.g., asthma or sleep apnea), or a biometric tool for authentication purposes,^[1–3]^ while skin RH variation patterns can be employed to assess human physiological and psychological parameters.^[3]^ In these examples, humidity is a useful bio-marker that needs monitoring at ambient and physiological conditions. However, humidity is often seen as an undesirable confounding factor in sensing of gases and volatile organic compounds (VOCs). Diseases as diabetes and cancer are associated with the presence of specific VOC bio-markers in exhaled air.^[2,4]^ As such, one of the most challenging goals in gas sensing and artificial olfaction is the detection and measurement of VOCs in ambient conditions where humidity is ubiquitous. Metal oxide semiconductors (MOS) and conducting polymers, the most standardized and used gas sensing materials, are greatly affected by humidity as it causes signal variations due to water cross-reactivity.^[5–8]^ To avoid humidity interference, gas sensors and electronic noses (e-nose systems) employ several mitigation measures, namely the use of nitrogen or dry air as a sample carrier gas,^[9,10]^ sample preconditioning chambers to control humidity content of the sample before sensor delivery,^[8]^ coating sensing materials with hydrophobic layers,^[11]^ or the employment of algorithms to correct the sensor’s signals based on humidity conditions.^[12,13]^ Another option lies in the search for alternative gas sensing materials where humidity monitoring and interference can be easily tailored and controlled.

Ionogels and derived materials represent a versatile class of transparent, flexible, mechanically stable, and durable sensors being considered excellent ionic skins as central components in wearable electronics, human-machine systems, implantable devices and soft robotics.^[14]^ In gas sensing, ionogels are also considered ideal sensors due to their stability in ambient conditions and tunable selectivity conferred by the ionic liquid.^[15,16]^ Ionic liquids play a key role in these properties due to their intrinsic high chemical diversity, low melting points, high chemical and thermal stability, very low volatility, intrinsic ionic conductivity, wide electrochemical window, high viscosity, high polarity, and good solvation properties.^[17]^

**Figure 1:**
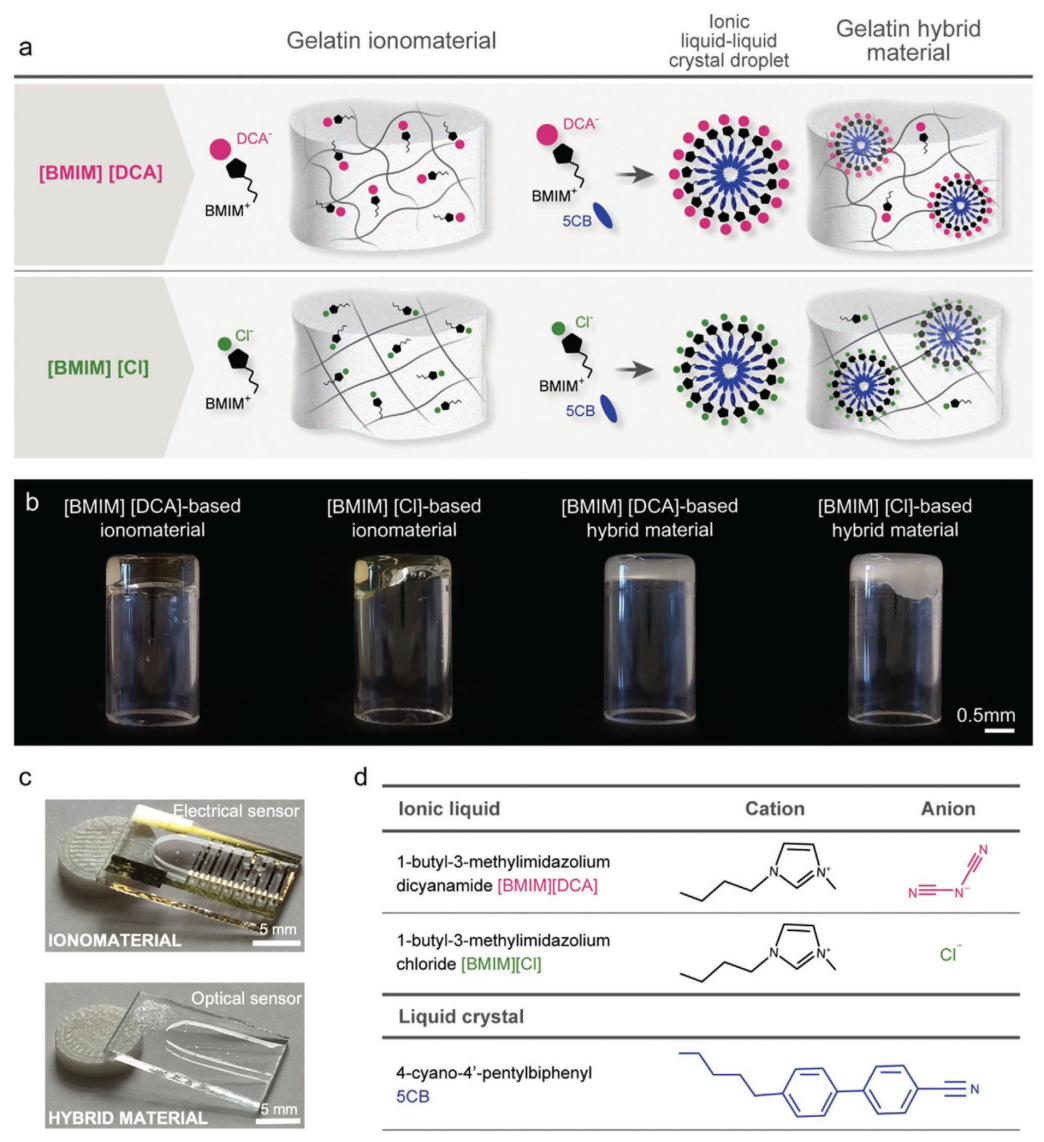
[BMIM][DCA] and [BMIM][Cl] gelatin ionomaterials and hybrid materials. a) Schematic representation. b) Macroscopic images of the ionomaterials and the hybrid materials and c) derived thin films used as electrical and optical sensors. d) Chemical structures of the ionic liquids and the liquid crystal used in this study

It is known that in 1-butyl-3-methylimidazolium-based ionic liquids, [BMIM][X], the anion [X]^−^ plays a dominant role on the interactions with water.^[18,19]^ Thus, we hypothesized that the effect of humidity in gas sensing materials containing ionic liquids with the 1-butyl-3-methylimidazolium ([BMIM]+) cation could be tailored by the choice of the ionic liquid anion. The anions dicyanamide ([DCA]^−^) and chloride ([Cl]^−^) were chosen due to their wide availability and different hydrophilicity and hydrogen bonding acceptor properties.^[19–21]^ To show the effect of the anion, in this work, we used gelatin formulations in ionic liquid, named as ionomaterials, and gelatin formulations in ionic liquid-incorporating liquid crystal (4-cyano-4′-pentylbiphenyl, 5CB) compartments, named as hybrid materials^[15]^ (Figure 1). In the first part of this work, we focused on the electrical response of the ionomaterials, while in the second part, we added the liquid crystal component to the formulation and investigated the optical response of the hybrid materials. In both cases, we investigated the electrical and optical responses of the materials firstly to different RH variations and then to model VOCs in dry (0% RH) and humid (50% RH) conditions. Our findings show the potential and versatility of ionomaterials and hybrid materials as designer humidity and VOC-responsive soft materials, with implications in artificial olfaction, wearable electronics and soft robotics.

## 2 Results and Discussion

### 2.1 Gelatin Ionomaterials and the Influence of Relative Humidity

Gelatin is a well-known gelling biopolymer with a typical repetitive sequence Ala-Gly-Pro-Arg-Gly-Glu-4Hyp-Gly-Pro, where Hyp is hydroxyproline.^[22]^ It derives from the thermal treatment of collagen that denatures collagen′s triple-helical structure originating individual soluble chains and small fragments. The triple helical structure of collagen is partially recovered when gelatin dissolved in hot water is cooled down. The re-formed segments of triple helices serve as "junction zones" or physical crosslinks between the gelatin polypeptide chains, which lead to gelation and hydrogel formation.^[23]^ Like in collagen, the triple helices are stabilised by a network of hydrogen bonds, with direct H-bonds (when bridging N–H⋯O═C from neighbour backbone strands); water-mediated H-bonds (with and without the contribution of Hyp hydroxy groups); and less conventional C*α*-H⋯O═C between neighbour strands. Water is also considered an intrinsic component of collagen by creating ordered water networks between neighbouring triple helices.^[24]^

**Figure 2:**
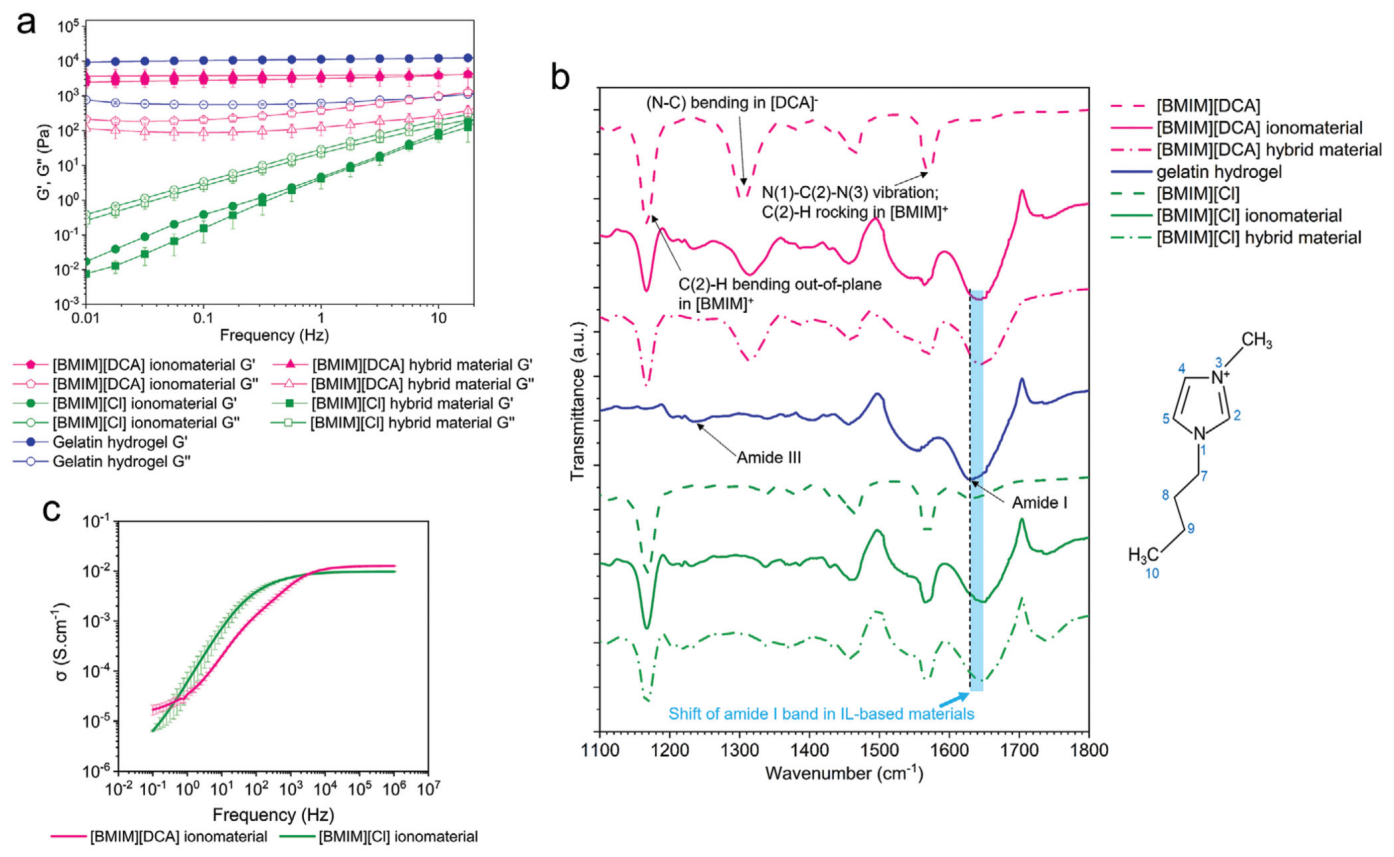
Characterization of gelatin ionomaterials and hybrid materials. a) Viscoelastic properties (shear storage (*G′*) and loss (*G″*) moduli) of hybrid materials composed of gelatin, 5CB, water, and either [BMIM][DCA] or [BMIM][Cl], respective ionomaterials and gelatin hydrogel (*n = 3*). b) Details of ATR-FTIR spectra in the amide region. The blue shaded area indicates the shift of the gelatin amide I band (C═O) to higher frequencies in the ionomaterials and hybrid materials compared to the hydrogel. The numbering of carbon and nitrogen atoms is represented near the [BMIM]+ structure to ease the interpretation of the spectra. c) Ionic conductivity of [BMIM][Cl]- and [BMIM][DCA]-based gelatin ionomaterials at room conditions (51% RH) (*n = 2*).

Ionic liquids can also dissolve gelatin giving raise to ionogels.^[22]^ In this work, the imidazolium-based ionic liquids [BMIM][DCA] and [BMIM][Cl] were studied to yield gelatin-ionomaterials. When cooled down, the gelatin/[BMIM][DCA]/water solution gave rise to self-supporting gels while gelatin/[BMIM][Cl]/water formulation remained a viscous fluid (Figure 1a,b). The viscoelastic properties of the prepared materials were assessed by rheometry, confirming the gel nature of [BMIM][DCA]-based ionomaterial (similar to gelatin hydrogel) and the viscous nature of the [BMIM][Cl]-based material suggesting entangled networks formed by topological interaction of the chains rather than crosslinking^[25]^ (Figure 2a).

Gelatin polypeptide chains assemble differently in water and in ionic liquids.^[15,22,26]^ X-ray scattering data (Figure S1, Supporting information) show that [BMIM][DCA] and [BMIM] [Cl] gelatin ionomaterials present the diffused arc at 4 Å (also visible in the hydrogel), typically associated with amorphous parts (polypeptide single chains in random coils configuration with no structural ordering or packing^[27]^), but lack the ring at 10.7 A characteristic in gelatin hydrogels due to triple helices’ assembly and order.^[15,22,28]^ The lack of structural ordering of polypeptide chains in ionomaterials is further confirmed by ATR-FTIR data, in which the amide I band of the gelatin hydrogel (1630 cm^−1^), representing C═O stretching of imide residues hydrogen bonded with water, is shifted to higher wavenumbers (1645–1647 cm^−1^), close to the vibration frequency of C═O groups in random coils^[29]^ (Figure 2c).

The [BMIM][DCA] and [BMIM][Cl] ionomaterials have the reduction of water content in common, when comparing with the control hydrogel. It has been proposed by several authors that physical crosslinking in gelatin ionomaterials results from hydrogen bonds, ionic and electrostatic interactions between gelatin polypeptide chains and ionic liquid ions.^[26]^ The imidazolium cation [BMIM]^+^, that is common in both ionomaterials, can establish ionic and electrostatic interactions with negatively charged amino acids, namely Glu and Asp. It is also a weak hydrogen bond donor, and as such can form hydrogen bonds with hydrogen bond acceptor groups in gelatin backbone and side chains. The cation [BMIM]^+^ has a short 4-carbon alkyl chain which may also establish hydrophobic interactions with hydrophobic amino acid residues (Leu, Ile, Met, and Val which represent about 17% of gelatin’s composition) and weak interactions with Pro.^[30]^ The ionic liquid anions can also establish ionic and electrostatic interactions with positively charged amino acid residues as Arg.^[30]^ However, these interactions alone cannot justify the difference between [BMIM][DCA] (gels) and [BMIM][Cl] (viscous fluids) ionomaterials, as these anions differ in volume, hydrogen bonding ability, and affinity to water. Several authors have shown that ionic liquid anions in particular have great impact on gelatin gelation.^[22,26,31]^ It is known that the [Cl]^−^ anion is hydrophilic and establishes very strong hydrogen bonds with water, acquiring a dense hydration layer of around 6 water molecules retained by strong O–H⋯Cl^−^ hydrogen bonds.^[19]^ The [Cl]^−^ anion is a stronger hydrogen bond acceptor than [DCA]^−^:^[32]^ according to the empirical polarity Kamlet-Taff parameters, the hydrogen bond acceptor ability (*β*) of [Cl]^−^ is 0.95 while that of [DCA]^−^ is 0.64, when associated with the [BMIM]+ cation.^[33]^ With this evidence, and in line with previous reports, the [Cl]^−^ anion will sequester water molecules contributing to the formation of water-rich and water-depleted compartments in the material, hence further reducing the amount of water molecules to mediate hydrogen bonds between polypeptide chains. In addition, as a strong H-bond acceptor, the [Cl]^−^ anion is known to break interstrand hydrogen bonds between neighbor backbone polypeptide chains. As a result, the lack of intermolecular bonding and the presence of water-depleted regions in the matrix limits the cohesion of the matrix, yielding the observable viscous iono-materials.^[34]^ Regarding the dicyanamide anion [DCA]^−^, it is considered less hydrophilic than [Cl]^−^, therefore establishing less interactions with water which becomes more available to interact with polypeptide chains. [DCA]^−^ has a boomerang-like structure, with a permanent dipole and varied resonance structures, being often used as a bridge ligand to form cross-linked and organized structures.^[35,36]^ So, physical crosslinking in [DCA]^−^ ionomaterials likely results from a combination of ionic and electrostatic interactions, dipole–dipole (for example with Gly) and hydrogen bonds with hydrogen bond donor groups in gelatin.^[37]^ Taken together, the properties of dicyanamide anion [DCA]^−^ promote physical cross-linking between gelatin chains and yield mechanically robust ionogels.

Although exhibiting different mechanical properties, both ionomaterials have shown to possess ionic conductivity (Figure 2b) due to the presence of ionic liquid molecules. Ionic conductivity is an intrinsic material property and represents the ability to conduct electric current due to the motion of ionic charge. Conductance is a measure of the ease with which an electric current passes through a material, being a result of the material conductivity and its geometry. To explore the gas sensing properties of the ionomaterials, we spread them as thin films on top of interdigitated electrodes (Figure 1c) which were then placed in an in-house assembled signal acquisition device (described in Supporting Information).^[22]^ Due to the ionic conductivity of the ionomaterials (Figure 2b), when an alternate voltage is applied to one of the interdigitated electrode terminals, it causes ions movement within the material, which generates an electrical current. The current is detected in the other electrode terminal and converted to a voltage value that is proportional to the conductance of the material. This is the baseline electrical signal of the ionomaterial at defined conditions. Adsorption and desorption of water or VOC molecules into the ionomaterial cause changes in charge mobility thus generating conductance variations relative to the baseline. This is the relative electrical signal of the ionomaterial.

The electrical response of [BMIM][DCA]- and [BMIM][Cl]-based ionomaterial thin films to variations of RH was studied by exposing the thin films to five humidification–drying cycles between 0% RH and one of four different RH levels (30%, 50%, 60%, and 70%, in independent experiments). The films were stored under controlled humidity (around 50-60% RH) during a maximum period of 1 week before being introduced in the sensors’ chamber and subjected to the cycles of RH variation. Prior to the first cycle, the sensors’ electrical signal was equilibrated during 15 minutes at 0% RH. Controlled levels of RH were generated using a nitrogen stream and a bubbler system with different saturated salt solutions (Figure S4, Supporting information).

**Figure 3:**
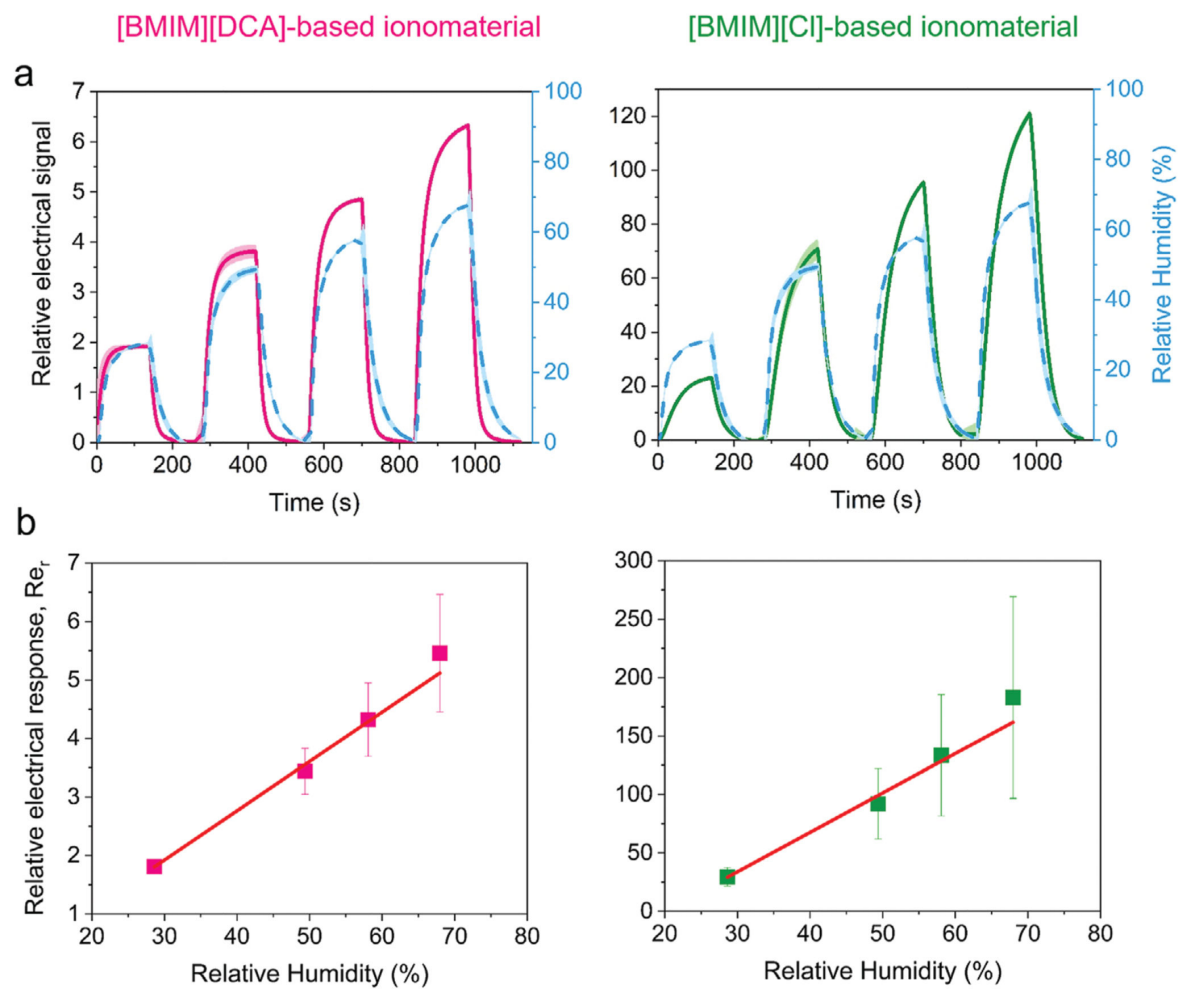
Analysis of the electrical response to humidity of [BMIM][DCA] and [BMIM][Cl] ionomaterial thin films. a) Relative electrical signal upon exposure humidification-drying cycles to increasing RH levels. The blue dashed line represents the variation of RH. The full lines and shadows represent, respectively, the average and standard deviation of 5 humidification–drying cycles to each RH level. b) Variation of the sensors relative electrical response (Re_r_) as a function of the RH level. For [BMIM][DCA] ionomaterials, Re_r_ = 0.084 *RH –* 0.607, R^2^ = 0.995 (*n* = 15) and for [BMIM][Cl] ionomaterials, Re_r_ = 3.373 *RH -* 67.395, *R*^2^ = 0.988 (*n* = 15).

Both gelatin and ionic liquids [BMIM][DCA] and [BMIM][Cl] are hydrophilic and water soluble. During humidification–drying cycles, water sorption and desorption into and from the ionomaterials occurred, inducing changes in the films’ relative electrical signal as shown in Figure 3a and Figure S2 (Supporting information). Upon humidification, an increase in ionomaterial films’ relative electrical signal was observed due to the sorption of water molecules to the ionomaterial. This effect is commonly attributed to an increase in the number of ion pathways through the material owing to a decrease of viscosity, leading to a higher ionic mobility, and, consequently, to a higher conductivity.^[22]^ Flushing with dry nitrogen promoted water desorption, decreasing ionomaterial films’ electrical signal values back to the baseline. Water sorption and desorption were very pronounced in [BMIM][Cl] ionomaterials with 15 μm thickness, which presented, a 47% (±15%) mass gain upon environment humidification (0% to 70% RH) and a 26% (±13%) mass loss upon subsequent drying to 0% RH. [BMIM][DCA] ionomaterials practically did not suffer mass changes detectable with the methodology used (in the order of 5%) (Figure S14a, Supporting information).

The dynamic response of [BMIM][DCA]- and [BMIM][Cl]-based gelatin ionomaterial thin films to the different humidified environments was shown to be reproducible along the five humidification–drying cycles, regardless of the relative humidity value tested (Figure 3a; Figure S2, Supporting information), suggesting that water vapor molecules interaction with the ionomaterial is a reversible and repeatable process.

The relative electrical response (Re_r_) of both sensor films (i.e., the maximum of the relative electrical signal within a humidification–drying cycle) linearly increases with the increase of RH between 30–70% RH values (Figure 3). However, the linear fitting parameters are very different (Figure 3b): the slope coefficient is 0.084 for [BMIM][DCA] ionomaterial and 3.373 for [BMIM][Cl] ionomaterial while the offset is 0.607 for the first and 67.395 for the latter. The slopes represent the sensitivity of the sensor, i.e., the sensor response variation per unit change in %RH.^[38]^ The ionomaterial with [BMIM][Cl] is 40 times more sensitive to RH changes than the ionomaterial with [BMIM][DCA]. This is attributed to its fluid and highly hygroscopic character. The offsets mean that for 0% RH, the sensing response is different from zero (as it corresponds to the baseline for each material), which has no implications in humidity sensing, as the signal-RH conversion function can be corrected to have a zero value to 0% RH.

Unlike [BMIM][DCA] ionomaterials, [BMIM][Cl] ionomaterials did not reach an equilibrium state during humidification period (Figure 3a; Figure S2, Supporting information), resulting in a higher response time, 80𠄣100 s, when compared with [BMIM][DCA], 40𠄣60 s. In both cases, the response to humidification is independent from the sensor history, as after 15 min in a dry environment (equilibration time before the first cycle), the relative electrical signal to a humidification step is identical in shape and response time to that yielded after short drying periods, of 140 s (subsequent 4 cycles). The RH level had an impact on the response times, though. It was possible to observe a 50% increase in the response time with the increase of RH from 30% to 70%. On the contrary, recovery times did not show significant differences for the different humidity values. [BMIM][DCA] thin films presented shorter recovery times than [BMIM][Cl] ones, 40 and 60 s respectively (Figure S3, Supporting information).

In accordance with previous work,^[1]^ these results show that the responsiveness to humidity exhibited by the ionomaterial sensors can be tuned by adjusting the anion of the ionic liquid.

### 2.2 Gelatin Ionomaterials and VOC Sensing Ability in Dry and Humidified Conditions

The capability of VOC sensing under dried (0% RH) and humidified (50% RH) conditions was further studied by exposing the ionomaterial thin films to vapors of four distinct model solvents (see details in the Supporting Information and Figure S5 in the Supporting information). Ethanol, acetone, toluene and hexane were chosen as representative of chemical groups with distinct polarity and hydrophilicity^[15, 39]^ (Table S2). An equilibration period under 0% RH or 50% RH conditions was applied to the thin films prior to the VOC sensing experiment. A blank assay was also performed under the same humidity and nitrogen flow rate conditions but without the presence of VOC. Small changes in the thin films’ relative electrical response were observed for both ionomaterial formulations (Figure S6, Supporting information) due to slight variations in the humidity content on the exposure and recovery streams, inherent to the design of the gas delivery system (Figure S5, Supporting information).

**Figure 4:**
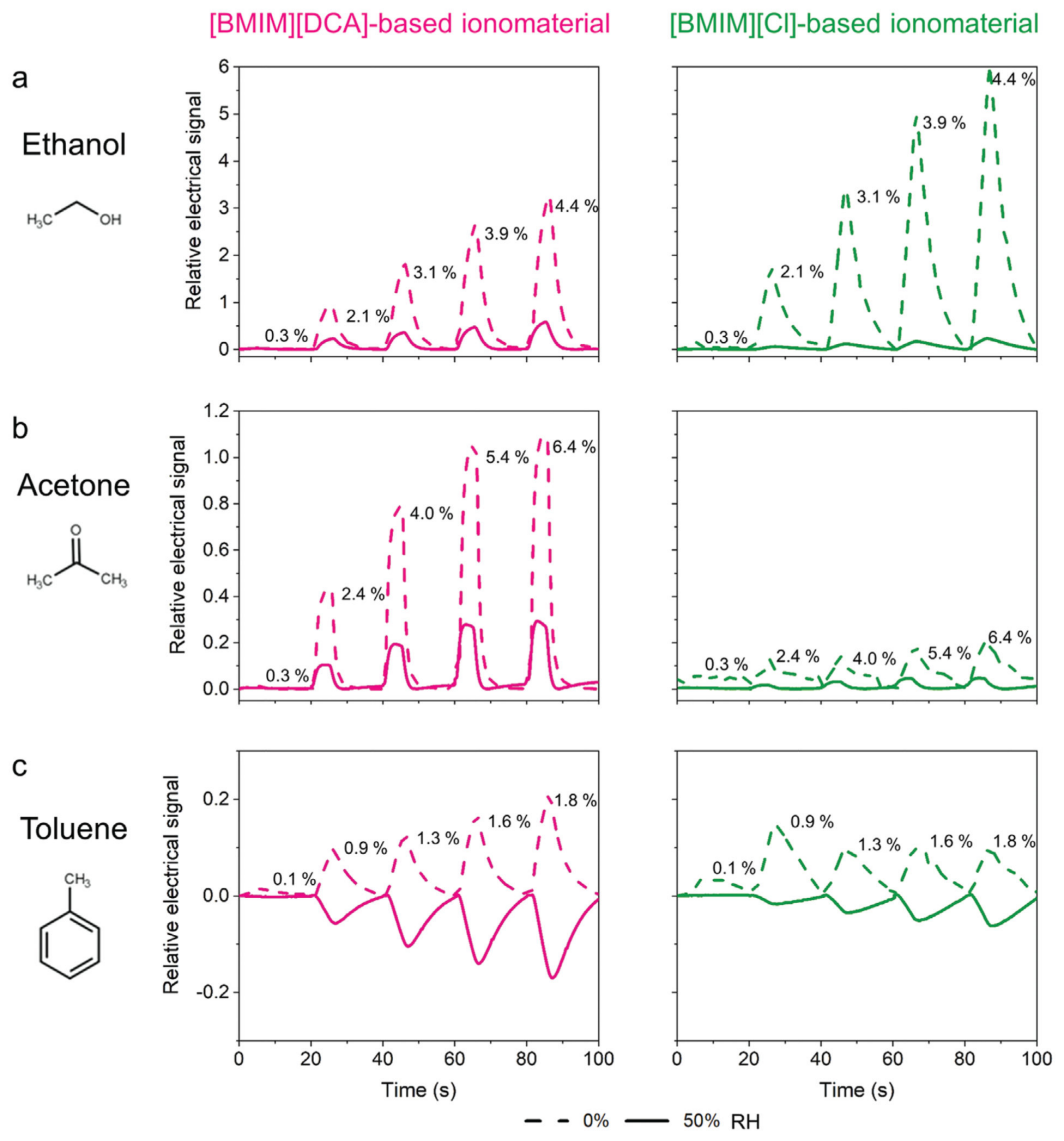
Relative electrical signal of gelatin ionomaterials with [BMIM][DCA] and [BMIM][Cl] upon exposure to increasing concentrations of ethanol, acetone, and toluene diluted in dry (0% RH, dashed line) or humidified (50% RH, full line) nitrogen. VOC concentrations (% (v/v)) are indicated for each condition. (*n = 5*).

In general, lower amplitude signals were obtained during VOC exposure at 50% RH (Figure 4; Figure S7, Supporting information) when compared with 0% RH. This is likely due to the competition between water molecules and VOCs toward components of the material. The [Cl]^−^ anion is a strong hydrogen bond acceptor. Ethanol and acetone are polar solvents with favorable adsorption in ionic liquids.^[40]^ Ethanol is also a protic and hydrogen bond donor. As such, hydrogen bond interactions between this VOC and the anion of the ionic liquid will be established. It was observed an exponential relationship (*R^2^* = 0.99) between the relative electrical response of [BMIM][DCA]- and [BMIM][Cl]-based ionomaterials and ethanol concentration (0.3–4.4%(v/v)) for both 0% and 50% RH (Figure S8, Tables S3 and S4, Supporting information). For the highest tested concentration, 4.4%(v/v), at 50% RH the relative response to ethanol was 7 times lower for [BMIM][DCA] ionomaterials and 17 times lower for [BMIM][Cl] ionomaterials than at 0% RH. Acetone is a hydrogen bond acceptor. In this case, hydrogen bond interactions will occur between acetone and the acidic hydrogen atoms of the ionic liquid’s cation, [BMIM]+. This interaction can be strengthened if the ionic liquid’s anion possesses dispersed charge like in [DCA]^−^,^[41]^ increasing acetone solubility in the ionic liquid. When exposed to acetone, the largest variations of the relative electrical response were observed for the ionomaterials with [BMIM] [DCA], where, at the highest tested concentration (6.4% (v/v)), the relative responses were approximately 3 times higher than those of [BMIM][Cl]-based ionomaterials (Figure S7, Supporting information). Although the ionomaterials’ relative responses linearly increased (*R*^2^ = 0.94 to *R^2^* = 0.99) within the tested VOCs concentrations (0.3–6.4%(v/v)) for the two studied ionomaterial formulations, for [BMIM][DCA] ionomaterials saturation was observed at 5.4%(v/v). Additionally, acetone detection limit was determined for [BMIM][DCA] ionomaterial thin film at 50% RH, 0.6%(v/v) (Figure S7, Tables S3 and S4, Supporting information).

Regarding the apolar and hydrophobic VOCs (hexane and toluene), different results were obtained. Hexane did not change the relative electrical response of both ionomaterials, as there were no differences between the blank and hexane signals (Figure S9, Supporting information). Due to its hydrophobic character, hexane hardly interacts with gelatin. Interactions with the ionic liquids are also not expected, as reported by Gonzalez-Miquel and co-workers, who used COSMO-RS (a computational method based on unimolecular quantum chemistry calculations^[42]^) to predict unfavorable interactions between hexane and ionic liquids with small imidazolium cations and polar anions due to repulsive electrostatic-misfit interactions.^[40]^

Lastly, [BMIM][DCA] and [BMIM][Cl] ionomaterial thin films were able to detect toluene but only in dry conditions. At 50% RH, the relative electrical signals were identical to those of the blank assay (Figure S9, Supporting information), suggesting that the interactions of water with the ionomaterials are prevalent compared to those of toluene. Toluene can establish π–π interactions with the imidazolium ring of ionic liquid’s cation, [BMIM]+. The weaker the electrostatic interaction between ionic liquid’s cation and anion is, the higher the free volume in the bulk ionic liquid and, consequently, more toluene molecules can be adsorbed onto the material.^[40]^ In a dry environment these interactions are probably the cause of the ionomaterials’ relative response variations. The relative response of [BMIM] [DCA] ionomaterial thin films to toluene at 0% RH linearly increased (*R*^2^ = 0.97) in the range of tested concentrations (0.1–1.8%(v/v)). On the other hand, the relative response of [BMIM][Cl] ionomaterial thin films stabilized, showing saturation for 0.1%(v/v) (Figures S7 and S8 and Tables S3 and S4, Supporting information).

In summary, the composition of the ionic liquids has a high impact on the molecular interactions of the ionomaterial with VOC molecules in humid and dry environments. The high hygroscopic character of [Cl]^−^-based thin films results in higher fluidity and higher conductivity changes with the increase of RH than the ionomaterial with [BMIM][DCA]. This pronounced influence of water molecules further restricts the VOC sensing ability of [BMIM][Cl] ionomaterials to dry conditions. On the other hand, [DCA]^−^ films, which are less responsive to humidity, have shown to be suitable for VOC sensing in both dry and humid conditions.

The response times of our electrical system to VOCs are in the order of 0.02–5 s, which are comparable to other works performed with different gelatin-based ionomaterials,^[39,43]^ where response times of 5 s were obtained when the materials were exposed to similar VOCs to those tested in our work (e.g., acetone, ethanol, hexane toluene). This is a very fast response compared to that reported recently metal oxide gas sensors, that can range between 30 and 80 s^[44]^ or even take a few minutes to respond^[45]^ to ethanol.

### 2.3 Gelatin Hybrid Materials and the Influence of Relative Humidity

In hybrid materials, the ionic liquid and liquid crystal molecules self-assemble into ionic liquid–liquid crystal spherical droplets entrapped in the gelatin ionomaterial matrix.^[15]^ Hybrid materials have mechanical properties identical to the corresponding ionomaterials: the [BMIM][DCA] hybrid material yields a self-supporting material whereas the [BMIM][Cl]-based formulation yields viscous materials (Figures 1 and 2a). ATR-FTIR spectra are also similar to those of the corresponding ionomaterials (Figure 2b), suggesting that the presence of the liquid crystal does not affect the molecular interactions established during the material production.

**Figure 5:**
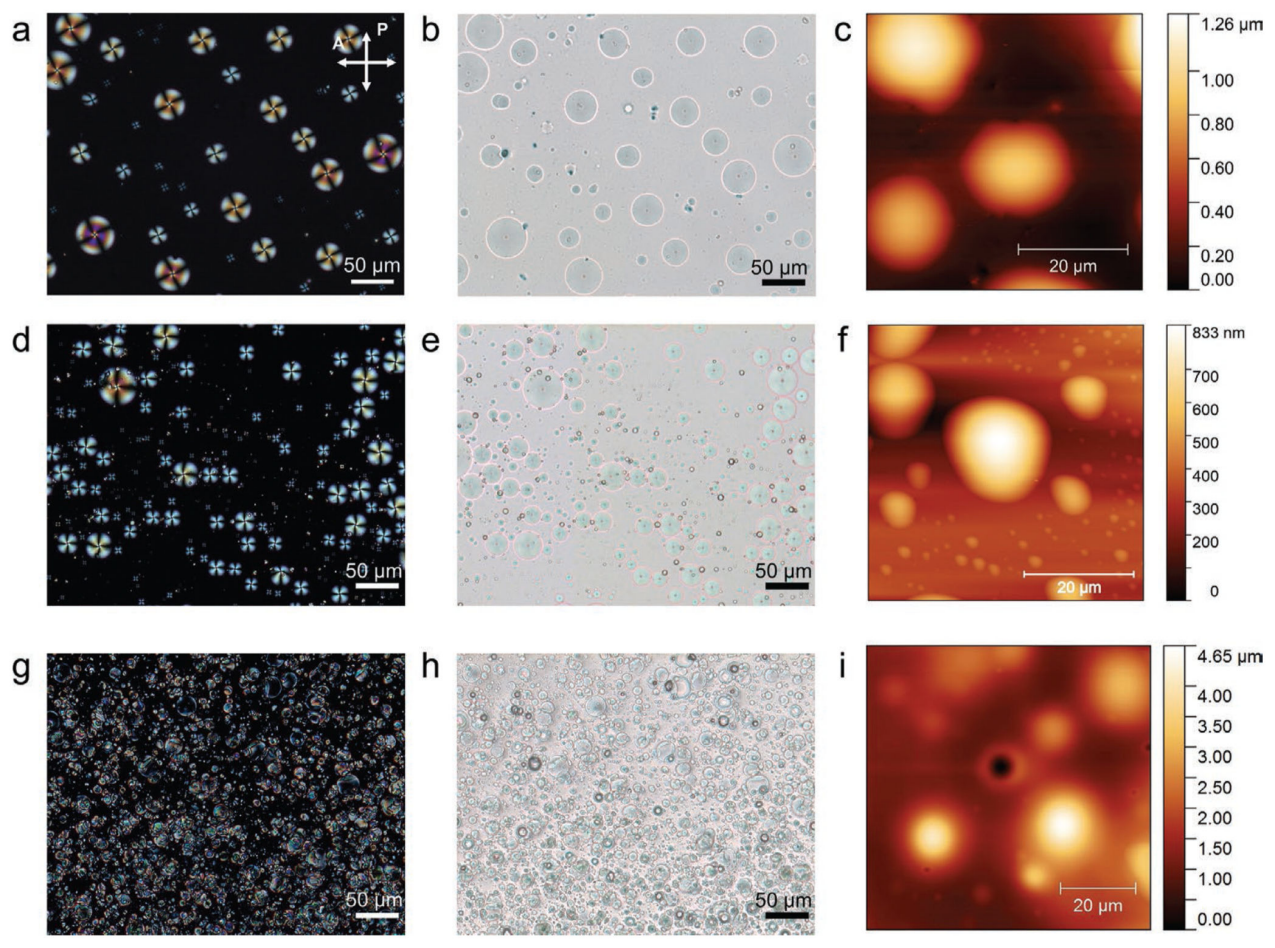
Morphological characterization of thin films of hybrid materials and control material without ionic liquid. a–c) Hybrid gel with [BMIM][DCA]. d–f) Hybrid material with [BMIM][Cl]. g–i) Control material without ionic liquid. a,d,g) Polarizing optical microscopy (POM) images with crossed polarizers. b,e,h) Bright-field optical microscopy images corresponding to the films’ regions in POM images. c,f,i) Topology of the films by atomic force microscopy.

Hybrid materials possess interesting optical properties given by the presence of liquid crystal molecules which can alter the polarisation of light, due to their birefringence. When 5CB molecules are confined in specific geometries, they adopt certain orientations, forming typical optical textures that can be observed with polarizing optical microscopy (POM).^[46]^ In hybrid materials, 5CB molecules are encapsulated in droplets (spherical interfaces formed by ionic liquid molecules) embedded in the ionomaterial matrix (Figure 5c,f). When the prepared hybrid materials were observed under POM, irrespective of the used ionic liquid, the droplets exhibited optical textures of Maltese crosses (Figure 5), which are characteristic of homeotropic anchoring of the liquid crystal to the spherical interfaces,^[46]^ where the liquid crystal molecules are oriented perpendicularly to the droplet interface, in a radial configuration. As the optical textures are identical in the presence of both [BMIM][DCA] and [BMIM][Cl], we conclude that the anion does not affect the orientation of the liquid crystal molecules in the droplets. Based on these observations and on the fact that, in the absence of ionic liquid, 5CB droplets in gelatin hydromaterials exhibit a bipolar configuration (Figure 5g,h), we can infer that in the hybrid materials, the [BMIM]+ cation is the main responsible for the homeotropic anchoring of liquid crystal molecules. It is likely that this anchoring is promoted by hydrophobic interactions between the alkyl chains of [BMIM]^+^ and those of 5CB.^[47]^ Other interactions contribute to stabilize the droplet interface. Namely, the imidazolium headgroup of [BMIM]^+^ can establish electrostatic interactions with the cyano group of 5CB. Also, both [DCA]^−^ and [Cl]^−^ anions can participate in hydrogen bonds and electrostatic interactions with the imidazolium headgroup of [BMIM]+,^[48]^ water molecules and gelatin matrix at the interface of the droplet.

The distinct composition of the droplet interface in [BMIM] [DCA] and [BMIM][Cl] hybrid materials had an impact on the droplets’ diameter, likely due to the different physical constraints created by [Cl]^−^ and [DCA]^−^ when interacting with the [BMIM]^+^ imidazolium headgroup. The nitrile and chloride anions have similar ionic radius (191 and 181 pm, respectively).^[49]^ As [DCA]^−^ contains two nitrile groups, it may cause steric hindrance at the liquid crystal droplet interface, thus promoting the assembly of larger droplets (33 ± 8 μm) than those formed with [Cl]^−^ (13 ± 4 μm). Due to these constraints, the hybrid materials made with [BMIM][DCA] usually exhibit a smaller droplet population (2 droplets/100 μm^2^) than those made with [BMIM][Cl] (19 droplets/100 μm^2^).

The ordering and phase transitions of the liquid crystal upon interaction of gas molecules with a hybrid material thin film result in changes in the intensity of polarized light transmitted through the film. The optical signal is inversely proportional to this light intensity. Details on the optical signal acquisition method and device can be found in our previous publications^[15,50,51]^ and in SI (Figure S19, Supporting information). To study the impact of humidity on the optical response of the hybrid material thin films, we coupled the optical signal acquisition device to the humidity delivery setup employed previously in the study of the electrical signal (Figure S4, Supporting information).

**Figure 6:**
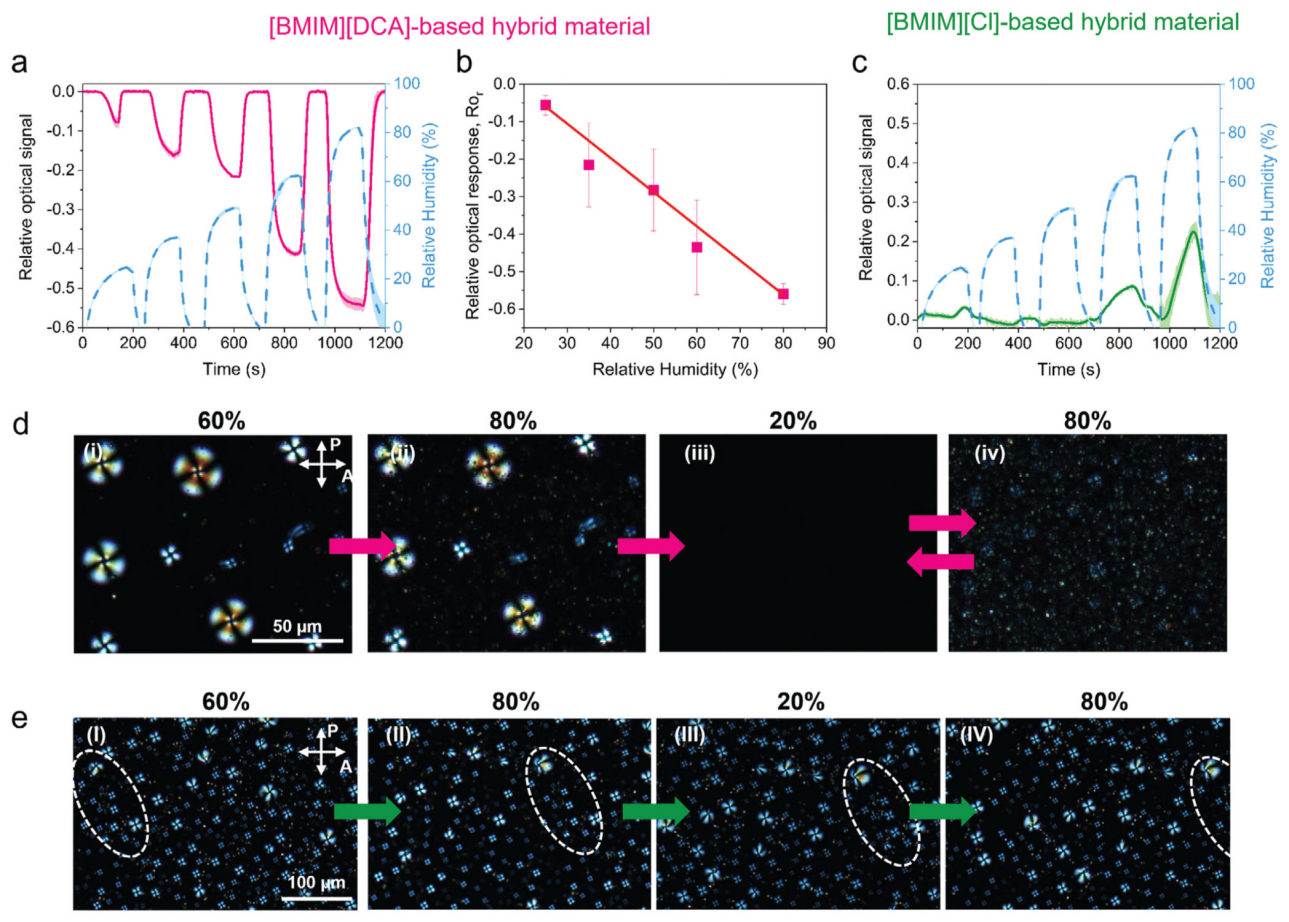
Analysis of the optical response to humidity of [BMIM][DCA] and [BMIM][Cl] hybrid material thin films. a,c) Relative optical signal upon exposure to humidification–drying cycles with increasing RH levels. The blue dashed line represents the variation of RH. The full lines and shadows represent, respectively, the average and standard deviation of 5 humidification-drying cycles to each RH level. b) Variation of the relative optical response (Ro_r_) as a function of the RH level (*n* = 12), Ro_r_ = -0.009 RH + 0.168, *R*^2^ = 0.996. d) POM images with crossed polarizers of a [BMIM][DCA] hybrid film during sequential exposure to humid nitrogen with RH varying between: i) 60% (room conditions), ii) 80%, iii) 20%, and iv) 80%. e) POM images with crossed polarizers of a [BMIM][Cl] hybrid material film during sequential exposure to humid nitrogen with RH varying between: I) 60% (room conditions), II) 80%, III) 20%, and IV) 80%; the dashed circles represent movement of the droplets.

The optical response of hybrid materials with [BMIM][DCA] and [BMIM][Cl] from 0% RH to five different RHs (25%, 35%, 50%, 60%, and 80%) is shown in Figure 6a,c and Figure S10 (Supporting information). It is evident that the anion of the ionic liquid has an impact on the optical response of the hybrid materials to humidity variations as response patterns with opposite behavior were obtained. Upon humidification, the optical signal of [BMIM][DCA] hybrid films decreases, while the signal of [BMIM][Cl] hybrid films increases. The reverse is observed for the drying period (Figure 6a,c). This suggests that the mechanism of water interaction with hybrid material components in the two formulations is distinct.

[BMIM][DCA] hybrid materials yield a repeatable optical signal waveform when varying the RH level from dry to humid conditions, indicating that water sorption is reversible (Figure S10, Supporting information). The relative optical response linearly increases with the increase of RH values (*R*^2^ = 0.996) (Figure 6b) while the response times decrease linearly (*R*^2^ = 0.997), reaching 40 s for the 0–80% RH change (Figure S11b, Supporting information). The opposite behavior is observed for the recovery times. [BMIM][Cl] hybrid materials are only sensitive to large RH variations (0–60% or 0–80%), yielding a signal that does not stabilize within the timeframe of the experiments (Figure 6a; Figure S10, Supporting information) and is 4 to 3 times slower than the corresponding signal of [BMIM][DCA] hybrid materials (Figure S11b,c, Supporting information). Thus, [BMIM][Cl]-based hybrid films are not very efficient as optical humidity sensors.

To understand the differences observed between the two hybrid materials, the changes in liquid crystal order and in the gelatin ionogel matrix during a humidification and drying cycle were monitored by POM (Figure 6d,e). The intensity of light transmitted through the films during this experiment was quantified as the average brightness of POM images (Figure S12a, Supporting information).

Our POM investigations showed that humidification of [BMIM][DCA] hybrid material films above room conditions (ranging from roughly 60% RH to 80% RH) has a minimal impact on the liquid crystal droplets morphology (Figure 6di,ii), leading to a minimal change in the film’s brightness (Figure S12, Supporting information). Subsequently, exposing the hybrid material to drier environment (20% RH) promotes a gradual liquid crystal phase transition from the nematic to the isotropic phase (Figure 6dii,iii), and thus the brightness of the film decreases until it reaches a minimum value (Figure S12, Supporting information). Due to the low hydration ability of [DCA]^−,[21,52]^ water molecules associated with [DCA]^−^ anions at the droplets interfaces easily desorb during drying. We hypothesize that, in the absence of water, most [BMIM][DCA] molecules become miscible with 5CB and consequently enter the droplets as an impurity thus lowering the clearing temperature of the liquid crystal and driving 5CB to isotropization. It is also plausible that some [BMIM][DCA] molecules migrate to the gelatin matrix. In both scenarios, the ionic liquid interface is disrupted and a partial leakage of isotropic liquid crystal from the droplets to the matrix (Figure S13a, Supporting information) is observed. This is concomitant with alterations in droplet volume and shape, unraveling spherical capsules imprinted in the matrix (Figure S13a,b, Supporting information) that possibly formed during gelation in presence of the initial liquid crystal droplet templates. Upon subsequent humidification from 20% to 80% RH ((iii) to (iv) in Figure 6d), the ionic liquid hydrates again (now immiscible with 5CB) leading to a fast reorganization of the material’s compartments, albeit leading to a morphology that is different from the initially observed. The liquid crystal and [BMIM][DCA] molecules that leaked to the matrix during the drying period self-assemble again, forming, this time, multiple small radial liquid crystal droplets that densely fill the matrix (diameter: 7 ± 3 μm; 13 droplets/100 μm^2^) ((iv) in Figure 6d). In subsequent cycles of drying and humidification this "new" film morphology is maintained, yielding a repeatable optical signal waveform, as seen in Figure S10 in the Supporting information. Similar morphology changes are expected during the first cycle for the other RH levels tested (0%–25%, 35%, 50%, 60%) due to relative instability of the interface of liquid crystal droplets in [BMIM][DCA]-based materials. The information given by the POM studies indicate that the [BMIM] [DCA] hybrid materials can be used as optical humidity sensors after an initial drying process to originate the film morphology that is reversible upon drying–humidification steps.

Regarding [BMIM][Cl] hybrid materials, POM images show that when these are alternately exposed to humidification (80% RH) and drying (20% RH) periods, the liquid crystal droplets preserve the radial configuration (Figure 6e). Despite flowing in the ionomaterial matrix and contacting with each other, droplet coalescence events are rare, which indicates that the ionic liquid-liquid crystal interface is more stable than in the [BMIM][DCA] hybrid materials. As [BMIM][Cl] is very hygroscopic^[20,53]^ and the [Cl]^−^ anion is a strong hydrogen bond acceptor,^[19]^ the droplet interface facing the matrix is probably stabilized by a hydration shell strongly bound to [Cl]^−^ anions. Thus, the optical signal collected from [BMIM][Cl] hybrid films in the signal acquisition device (Figure 6c), is practically insensitive to humidity. The slight variations observed in the films’ brightness and optical signal could be attributed to the movement of droplets in the films and to films’ swelling and contracting upon water sorption and desorption, respectively (Figures S12 and S14, Supporting information). Indeed, when the environment of [BMIM][Cl] hybrid films with 30 μm thickness changed from dry (0% RH) to humid (85% RH), a 24% (±2%) mass increase by water sorption was observed. With subsequent drying to 0% RH, the material loses water, resulting in a similar (21 ± 2%) mass loss. [BMIM][DCA] hybrid films present much lower mass variations, in the order of 9% (±1%).

Similar to the electrical signals (Figure 3a), the optical signal does not stabilize as a consequence of [BMIM][Cl] hybrid materials gaining and losing water without reaching an equilibrium state within the time frame of the experiments. When the films are exposed to dryer environments than the RH at which the films were stored previously to use (25%, 35% and 48% RH), no optical response is obtained (Figure 6c; Figure S12, Supporting information). The preferential interaction of humidity with the gelatin matrix rather than with [BMIM][Cl] could be confirmed by investigating the response of a control film prepared with gelatin, water and liquid crystal when subjected to similar variations of humidity. No liquid crystal re-ordering or phase transitions were observed (Figure S15, Supporting information). However, the film brightness and optical signal present slight variations, comparable to those exhibited by the [BMIM][Cl] hybrid films.

As a conclusion, the optical changes in [BMIM][DCA] hybrid films to humidity are predominantly driven by the liquid crystal ordering and phase transitions due to droplet interface instability. In [BMIM][Cl] hybrid films the existence of a stable hydration layer at the droplets surface results in a response profile to varying RH limited by the kinetics of water sorption-desorption and therefore is less intense and much slower. Matrix swelling and contraction occur in both hybrid films (Figure S14, Supporting information), but they are less important to the optical signal in [BMIM][DCA] hybrid films because liquid crystal ordering and phase transitions have a predominant contribution.

### 2.4 Gelatin Hybrid Materials and VOC Sensing Ability in Dry and Humidified Conditions

**Figure 7:**
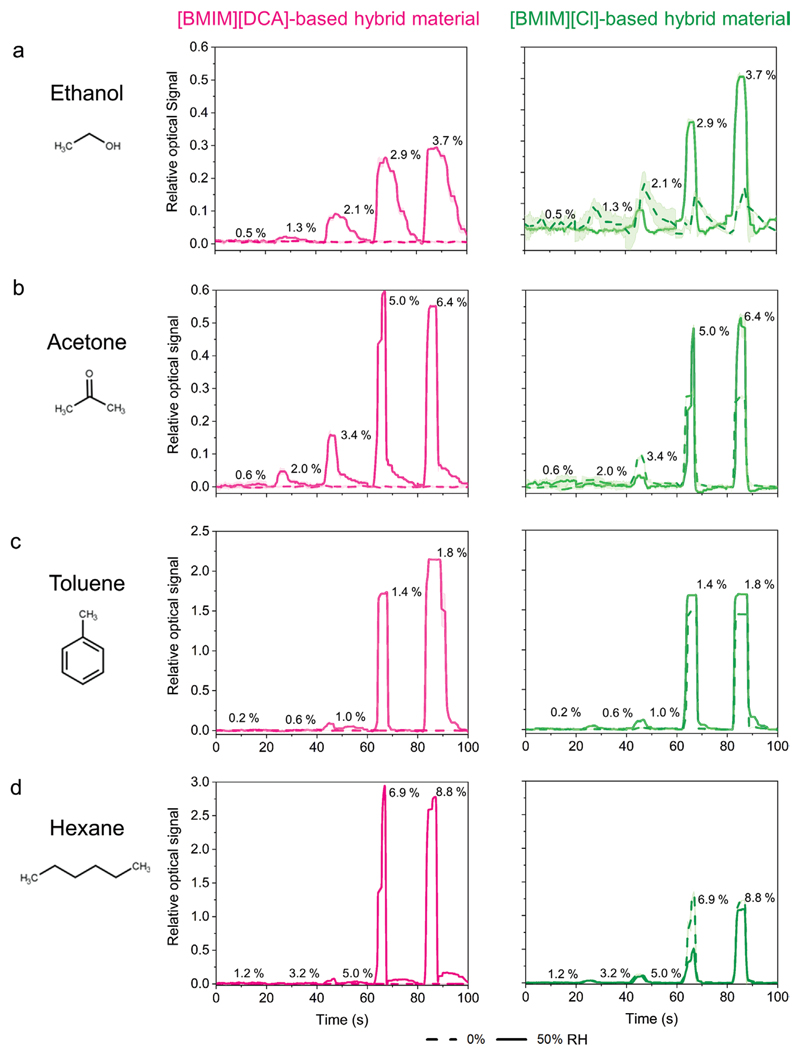
Variations of [BMIM][DCA] and [BMIM][Cl] hybrid material thin films’ relative optical signal to increase concentration of ethanol, acetone, toluene and hexane under dried (0% RH) and humidified (50% RH) environmental conditions (*n = 5*). VOC concentrations (% (v/v)) tested are indicated for each response cycle.

Contrary to what happens with the electrical sensors (Figure 4; Figure S7, Supporting information), the presence of 50% RH in the VOC samples improves sensing performance of [BMIM] [DCA] hybrid material films (Figure 7; Figure S16, Supporting information). This is because, as we concluded from our POM studies, the presence of water in the hybrid material is critical to maintain the radial configuration of the liquid crystal. In absence of humidity, the liquid crystal in [BMIM][DCA] hybrid materials is isotropic and the optical probes are not functional, thus hampering the VOC sensing property. This also agrees with the observation, in section 2.3., that below 20% RH, the optical signal of [BMIM][DCA] hybrid films is saturated in the upper limit of the scale (Figure 6a), which corresponds to the isotropic state of all liquid crystal droplets due to water depletion. At 50% RH, VOC presence can be detected through changes in brightness of the films caused by the disruption of the liquid crystal order upon VOC sorption to the material. Regarding [BMIM][Cl] hybrid materials, as seen in section 2.3., radial liquid crystal droplets are present, independently of the RH, and they can sense VOCs in both dry and humid conditions (Figure 7; Figure S16, Supporting information). Tables S5–S7 summarize the findings.

A blank assay identical to the performed for the electrical signal study was carried out. [BMIM][Cl] hybrid materials did not respond to small RH variations of the nitrogen stream in the absence of VOCs (Figure S17, Supporting information), as expected from the results of the previous section (Figure 6a). In turn, [BMIM][DCA] hybrid materials exhibited small responses. In the exposure stage the nitrogen stream is drier than in the recovery stage, which causes the [BMIM][DCA] hybrid materials films signal to increase and then decrease (Figure S17, Supporting information), in accordance with the results of the previous section (Figure 6a). Nonetheless, the relative amplitude of the responses in the blank assay are not significant (4 to 10 times lower) when compared to those yielded in the presence of VOC (Figure 7).

As it is a hydrogen bond donor, ethanol tends to interact preferentially with gelatin, water and the anion of the ionic liquids^[33]^ in the hybrid materials through hydrogen bonds. Direct interactions with the interface of the liquid crystal droplets are less probable. Thus, the optical signal of [BMIM][DCA] and [BMIM][Cl] hybrid films to ethanol is likely due to variations of fluidity of the matrix combined with slight disturbances of the liquid crystal order as ethanol interacts with the ionic liquid anions at the droplets interface. Therefore, the relative amplitudes of the responses are low, and increase with the concentration of ethanol (following a logistic regression) for both [BMIM][DCA] (*R*^2^ = 0.992) and [BMIM][Cl] (*R*^2^ = 0.999) hybrid materials at 50% RH. (Figures S16 and S18, Tables S5–S7, Supporting information). Limits of detection around 1% (v/v) were obtained for ethanol with both hybrid material formulations at 50% RH. For [BMIM][DCA] hybrid materials, a saturation concentration of 3.13% (v/v) was observed (Table S5 and S7). At 0% RH, [BMIM][Cl] hybrid materials did not yield a quantitative response.

Acetone, toluene, and hexane have distinct polarity characters. Due to the "domino effect" that characterizes the propagation of ordering perturbations through liquid crystal molecules, these VOCs cause a pronounced increase in the relative response of both sensor formulations after a certain triggering concentration, likely at which the number of VOC molecules becomes sufficient to reach the droplets interface and trigger the phase transition of more and more liquid crystal droplets (Figure 7; Figure S16, Supporting information). Before that concentration, the variation of optical response is minimal. Afterward, the variation is much larger. The triggering concentration depends on the VOC affinity to interact with the different components of the droplet and increases in the order toluene < acetone < hexane for both sensor formulations (Tables S5–S7, Supporting information). Toluene, due to its aromatic structure, can establish *π–π* interactions with the imidazolium ring headgroup of [BMIM]+, at the interface of the droplet. Acetone can also interact with the interface of the droplet, through hydrogen bonds with the acidic hydrogen of the imidazolium ring in [BMIM]+ and cation–dipole electrostatic interactions. Hexane, on the other hand, must enter the droplet to establish its preferential interactions (hydrophobic) with the alkyl chains of either [BMIM]^+^ or 5CB, which might require higher concentration of VOC.

The lowest detection limit was observed for toluene (0.53% (v/v)) with [BMIM][Cl] hybrid materials at 50% RH. The lower saturation limit was also observed for toluene, between 1.4 and 1.8% (v/v) for both hybrid material formulations. Hexane was the VOC detected up to larger concentrations (7–8% (v/v)) with the two hybrid material formulations. (Tables S5–S7, Supporting information).

Combining the above observations, we can conclude that the change of anion from [DCA]^−^ to [Cl]^−^ in the studied ionic liquid is crucial for the operation of the hybrid material sensors with dry VOC samples. When VOC samples are humid, there is not an evident outperformance of one sensor formulation over the other, given the similar response profiles, limits of detection and saturation concentrations to the tested VOCs.

The response times of our optical systems to VOCs are in the order of 3–5 s, which in fact are faster than most reported examples using liquid crystals.^[46]^ The response times to volatiles strongly depend on the format of the sensing system and the liquid crystal component. As an example, Shibaev et al.^[54]^ studied chiral liquid crystal (CLC) compositions with the selective reflection band in a visible spectral range for optical detection of various volatiles. It is a multi-element array and each element of the array is a droplet of CLC with a different composition. In the case of toluene vapours isotropization occurs after 3–4 min of exposure and in the case of ethanol isotropization starts only after 40 min of exposure.

## 3 Conclusions

An unmet challenge in artificial olfaction is the ability to develop gas sensors where the response to humidity is tuned and controlled by the sensing material itself. Here, this challenge is tackled with a simple design approach. Humidity sensing and humidity interference in VOC sensing is controlled through the design of gelatin-based ionomaterials and hybrid materials as electrical and optical sensors. In this design, the anions [DCA]^−^ and [Cl]^−^ of room temperature ionic liquids from the 1-butyl-3-methylimidazolium family, tailor the effect of humidity in the materials properties. We obtain either humidity-sensitive or humidity-tolerant VOC sensors that do not require sample preconditioning or signal processing for correction of humidity impact.

The electrical signal of gelatin ionomaterials thin films to the presence of gaseous analytes is associated with changes in ionic conductivity. Both [BMIM][DCA] and [BMIM][Cl] ionomaterial thin films are linearly responsive to increasing variations of RH (between 30–70% RH) due to the intrinsic hydrophilicity of the matrix as water increases the mobility of charge carriers. However, [BMIM][Cl] ionomaterials yield more intense responses (2 order of magnitude higher) that did not stabilize in the time course of the experiments due to strong hydrogen bond interactions between the [Cl]^−^ anion and water molecules. For this same reason, higher VOC sensing responses were obtained under dried environments (0% RH) as VOC interaction with ionic liquid cation or anion were weaker than those established with water. Regardless of the ionic liquid, the ionomaterial thin films’ sensing response increases with the increase of VOC molecules polarity.

The optical signal of the hybrid materials depends on the ordering and phase transitions of the liquid crystal molecules encapsulated by the ionic liquid in droplets. While the presence of the highly hydrophilic [Cl]^−^ anion increases sensitivity to humidity for the electrical response, for the optical signal, the reverse is observed. The [Cl]^−^ anion stabilizes the droplet interface and prevents water sorption and desorption from disturbing the ordered liquid crystal molecules, thus blocking humidity sensing properties. Thus, [BMIM][Cl] hybrid material thin films can detect and quantify all four tested VOCs in dry or room (50% RH) conditions, which represents a competitive advantage regarding most gas-sensor types, that require signal correction for cross-reactivity in humid environments. The less hydrophilic [DCA]^−^ anion, on the other hand, creates a more unstable droplet interface. When the materials are dry, the liquid crystal becomes isotropic, which hampers optical VOC sensing in dry environments with [BMIM][DCA] hybrid films.

The performance of hybrid materials as optical VOC sensors complements the performance of the corresponding ionomaterials electrical VOC sensors, as for example the ionomaterials could not detect hexane while the hybrid materials did.

In the future, the response function of both electrical and optical sensors for different VOCs at distinct RH values can be determined for well-defined applications and respective target VOCs. Typically, gas sensors operate in a wide range from ppb (or even ppt) to ppm VOC concentrations. For applications where VOC concentrations are higher, most current gas sensors require a sample dilution step,^[55]^ and there is in fact a lack of sensing solutions for environments with higher VOC concentrations.^[56]^ The presented ionomaterials and hybrid materials could fill this gap, as they operate in the high ppm to percent VOC concentration ranges. These environments are found in headspace samples from fermentation processes^[57]^ and quality control of alcoholic beverages^[58]^ or hand sanitizers,^[45]^ that could benefit from the use of non-invasive rapid or on-line sensing solutions.

Moreover, our electrical and optical systems presented lower response times to VOCs when compared with other reported sensors such as metal oxide sensors^[44,45]^ or liquid crystals.^[46]^

This work has established the design rules for the development of synergetic dual electro-optical transduction for humidity and VOC sensing. The simple variation of the ionic liquid anion in the formulation of the materials directly reflects in the performance of electrical and optical sensing of dry or humid samples, as well as in changes of VOC selectivity. There are innumerable ionic liquid compositions commercially available, and such a diversity extends the impact of the concept presented in this work, as tuning the anion and cation can give further improvements for sensing applications. The dual-mode opto-electrical sensing properties of the materials can be further explored regarding the complementarity of the signals and versatility to adapt to the sample conditions, offering potential to design sensor devices compatible with operation at room conditions, which are promising for innovative applications in gas sensing and artificial olfaction, namely when envisaging wearable and bionic devices.

## Supplementary Material

Supporting Information Supporting Information is available from the Wiley Online Library or from the author.

## Data Availability

The data that support the findings of this study are available from the corresponding author upon reasonable request.

## References

[1] Xiao S, Nie J, Tan R, Duan X, Ma J, Li Q, Wang T (2019). Mater Chem Front.

[2] Tai H, Wang S, Duan Z, Jiang Y (2020). Sens Actuators, B.

[3] He J, Xiao P, Shi J, Liang Y, Lu W, Chen Y, Wang W, Theato P, Kuo S, Chen T (2018). Chem Mater.

[4] Liu W, Xu L, Sheng K, Zhou X, Dong B, Lu G, Song H (2018). NPG Asia Mater.

[5] Hu W, Wan L, Jian Y, Ren C, Jin K, Su X, Bai X, Haick H, Yao M, Wu W (2018). Adv Mater Technol.

[6] Imam N, Cleland TA (2020). Nat Mach Intell.

[7] Ghanbarian M, Zeinali S, Mostafavi A (2018). Sens Actuators, B.

[8] Paknahad M, Bachhal JS, Hoorfar M (2018). Anal Chim Acta.

[9] Liu B, Huang Y, Kam KW, Cheung W-F, Zhao N, Zheng B (2019). Biosens Bioelectron: X.

[10] Gao A, Wang Y, Zhang D, He Y, Zhang L, Liu Y, Wang Y, Song H, Li T (2020). Sens Actuators, B.

[11] Deng Y, Sun J, Jin H, Khatib M, Li X, Wei Z, Wang F, Horev YD, Wu W, Haick H (2018). Adv Healthcare Mater.

[12] Huerta R, Mosqueiro T, Fonollosa J, Rulkov NF, Rodriguez-Lujan I (2016). Chemom Intell Lab Syst.

[13] Wei P, Ning Z, Ye S, Sun L, Yang F, Wong K, Westerdahl D, Louie P (2018). Sensors.

[14] Li T, Wang Y, Li S, Liu X, Sun J (2020). Adv Mater.

[15] Hussain A, Semeano ATS, Palma SICJ, Pina AS, Almeida J, Medrado BF, Pádua ACCS, Carvalho AL, Dionfsio M, Li RWC, Gamboa H (2017). Adv Funct Mater.

[16] Netto MMO, Gonçalves WB, Li RWC, Gruber J (2020). Sens Actuators, B.

[17] Silvester DS (2019). Curr Opin Electrochem.

[18] Huddleston JG, Visser AE, Reichert WM, Willauer HD, Broker GA, Rogers RD (2001). Green Chem.

[19] Fedotova MV, Kruchinin SE, Chuev GN (2017). J Mol Liq.

[20] Cao Y, Chen Y, Sun X, Zhang Z, Mu T (2012). Phys Chem Chem Phys.

[21] Sagawa N, Shikata T (2014). Phys Chem Chem Phys.

[22] Vidinha P, Lourenço NMT, Pinheiro C, Brás AR, Carvalho T, Santos-Silva T, Mukhopadhyay A, Romao MJ, Parola J, Dionisio M, Cabral JMS (2008). Chem Commun.

[23] de Wolf FA, Chapter V (2003). Prog Biotechnol.

[24] Bella J (2016). Biochem J.

[25] Picout DR, Ross-Murphy SB (2003). Sci World J.

[26] Lourengo NMT, Nunes AVM, Duarte CMM, Vidinha P (2011). Applications of Ionic Liquids in Science and Technology.

[27] Itoh M, Okawa Y, Kobayashi H, Ohno T, Okamotot Y, Katoh T (1994). J Photogr Sci.

[28] Bigi A, Panzavolta S, Rubini K (2004). Biomaterials.

[29] Prystupa DA, Donald AM (1996). Polym Gels Networks.

[30] Tomé LIN, Domínguez-Pérez M, Cláudio AFM, Freire MG, Marrucho IM, Oscar Cabeza OC, Coutinho JAP (2009). J Phys Chem B.

[31] Mcneice P, Zhao Y, Wang J, Donnelly GF, Marr P (2017). Green Chem.

[32] Zhou T, Chen L, Ye Y, Chen L, Qi Z, Freund H, Sundmacher K (2012). Ind Eng Chem Res.

[33] Lungwitz R, Friedrich M, Linert W, Spange S (2008). New J Chem.

[34] Rawat K, Pathak J, Bohidar HB (2014). Soft Matter.

[35] Kutasi AM, Batten SR, Moubaraki B, Murray KS (2002). J Chem Soc, Dalton Trans.

[36] Kliuikov A, Bukrynov O, Čižmár E, Váhovská L, Vitushkina S, Samofová E, Potočňák I (2021). New J Chem.

[37] Batista MLS, Kurnia KA, Pinho SP, Gomes JRB, Coutinho JAP (2015). J Phys Chem B.

[38] Güntner AT, Koren V, Chikkadi K, Righettoni M, Pratsinis SE (2016). ACS Sens.

[39] Carvalho T, Vidinha P, Vieira BR, Li RWC, Gruber J (2014). J Mater Chem C.

[40] Gonzalez-Miquel M, Palomar J, Rodriguez F (2013). J Phys Chem B.

[41] Ruiz E, Ferro VR, Palomar J, Ortega J, Rodríguez JJ (2013). J Phys Chem B.

[42] Klamt A, Jonas V, Bürger T, Lohrenz JCW (1998). J Phys Chem A.

[43] Gonçalves WB, Cervantes EP, Pádua ACCS, Santos G, Palma SICJ, Li RWC, Roque ACA, Gruber J (2021). Chemosensors.

[44] Cong S, Sugahara T, Wei T, Jiu J, Hirose Y, Nagao S, Suganuma K (2016).

[45] Güntner AT, Magro L, van den Broek J, Pratsinis SE (2021). iScience.

[46] Esteves C, Ramou E, Porteira ARP, Moura Barbosa AJ, Roque ACA (2020). Adv Opt Mater.

[47] Ramou E, Rebordāo G, Palma SICJ, Roque ACA (2021). Molecules.

[48] Hunt PA, Ashworth CR, Matthews RP (2015). Chem Soc Rev.

[49] Zhao H (2006). J Chem Technol Biotechnol.

[50] Esteves C, Santos GMC, Palma SICJ, Costa HMA, Alves VD, Porteira AR, Morais BM, Ferreira I, Gamboa H, Roque ACA (2019). Mater Today Bio.

[51] Santos G, Alves C, Pádua ACCS, Palma SICJ, Roque ACA, Roque A, Fred A, Gamboa H (2019).

[52] He F, Liu H, Xiong X, Zhai S (2018). J Adv Opt Photonics.

[53] Cao Y, Chen Y, Wang X, Mu T (2014). RSC Adv.

[54] Shibaev PV, Roslyak O, Plumitallo J, Gullatt E, Aparajita U (2020). Appl Phys A: Mater Sci Process.

[55] Luchner M, Gutmann R, Bayer K, Dunkl J, Hansel A, Herbig J, Singer W, Strobl F, Winkler K, Striedner G (2012). Biotechnol Bioeng.

[56] Potyrailo RA, Go S, Sexton D, Li X, Alkadi N, Kolmakov A, Amm B, St-pierre R, Scherer B, Nayeri M, Wu G (2020). Nat Electron.

[57] Feng Y, Tian X, Chen Y, Wang Z, Xia J, Qian J, Zhuang Y, Chu J (2021). Bioresour Bioprocess.

[58] Abegg S, Magro L, Van Den Broek J, Pratsinis SE, Guntner AT (2020). Nat Food.

