# Supplementary material for: Tackling Humidity with Designer Ionic Liquid-Based Gas Sensing Soft Materials
Source: Adv Mater. Author manuscript; Available in PMC 2022 Jul 12. (PMC7613046; doi:10.1002/adma.202107205)
Supplement: Supporting Information — is available from the Wiley Online Library or from the author. [file EMS146502-supplement-Supporting_Information.pdf]

ADVANCED MATERIALS

Supporting Information

for *Adv. Mater.,* DOI: 10.1002/adma.202107205

Tackling Humidity with Designer Ionic Liquid-Based Gas Sensing Soft Materials

*Carina Esteves, Susana I. C. J. Palma, Henrique M. A. Costa, Cláudia Alves, Gonçalo M. C. Santos, Efthymia Ramou, Ana Luísa Carvalho, Vitor Alves, and Ana C. A. Roque**

Supporting Information

Tackling Humidity with Designer Ionic Liquid-Based Gas Sensing Soft Materials

Carina Esteves[Ψ], Susana I.C.J. Palma[Ψ], Henrique M.A. Costa[Ψ], Cláudia Alves, Gonçalo M.C. Santos, Efthymia Ramou, Ana Luísa Carvalho, Vitor Alves, Ana C.A. Roque[*]

Detailed Experimental Section

Materials

The biopolymer gelatin (from bovine skin, gel strength ~225 Bloom, Type B) was purchased from Sigma-Aldrich (Portugal). The liquid crystal 4-cyano-4'-pentylbiphenyl (5CB, >98.0%) was purchased from TCI Europe (Belgium). The ionic liquids 1-butyl-3-methylimidazolium dicyanamide ([BMIM][DCA], >98.0%) and 1-butyl-3-methylimidazolium chloride ([BMIM][Cl], >98.0%) were purchased from IoLiTec (Germany). The anhydrous binary salts magnesium chloride (≥98.0%), potassium carbonate (99.9%) and sodium bromide (≥99.0%) were purchased from Sigma-Aldrich (Portugal), sodium chloride (>99.5%) was purchased from VWR Chemicals (Portugal). The organic solvent n-hexane (>95.0%) was purchased from Fisher Chemical (Portugal), acetone (>97.0%) was purchased from Sigma-Aldrich (Portugal), ethanol (96.0%) and toluene (99.5%) were purchased from Panreac AppliChem (Portugal).

Preparation of ionomaterial and hybrid material thin films

Hybrid materials were produced following the protocol described in [1–3]. The ionomaterials were prepared according with the same experimental procedure but without adding the liquid crystal component. The water content, determined by Karl Fischer titration was 21% and 20% for [BMIM][DCA] and [BMIM][Cl]-based ionomaterials, respectively.

Ionomaterial solutions were deposited onto a gold-titanium (60 nm-6 nm) interdigitated electrodes on glass substrate (18 parallel, 300 μm in width, spaced by 300 μm), using an automatic film applicator equipped with a heated bed and a quadruplex for a pre-defined thickness of 15 μm (TQC Sheen). Equally, hybrid material hot solutions were spread onto glass substrates but using the quadruplex with the pre-defined thickness of 30 μm.

The glass substrates were cleaned immediately before spreading the ionomaterial/hybrid material solution using the film applicator. The cleaning procedure consisted in immersion of the substrates in distilled water and then into isopropanol (10 minutes each solvent), followed by drying with compressed air.

Replicates (*n=3*) of each film formulation were used in the humidity and volatile organic compound (VOC) exposure experiments.

Attenuated Total Reflectance – Fourier-Transform Infrared Spectroscopy (ATR-FTIR)

A PerkinElmer Spectrum Two FTIR spectrometer with a $LiTaO_3$ / DTGS detector and an ATR accessory equipped with a ZnSe cell were used. Samples of ionomaterial, gelatin hydro material and hybrid films were prepared as described in the section above and transferred for the ATR cell for analysis. All measurements were made in the region between 400 – 4000 cm^{-1} with a resolution of 0.5 cm^{-1} and 40 scans at room temperature and an atmosphere of ambient air. The background spectrum of ambient air was subtracted from the samples spectra and the results were presented in transmittance (%) units.

Rheometry measurements

Samples of gelatin hydrogel and [BMIM][DCA] ionomaterial and hybrid material were prepared and left resting inside an O-ring mold with 20 mm inner diameter and 1 mm height to form a disk. The disk was placed in the rheometer plate. Since [BMIM][Cl]-based materials do not gelate, the samples (400 µl) were dispensed with a pipette directly to the rheometer plate. Rheological measurements were carried out using a controlled stress HAAKE MARSIII rheometer (Thermo Scientific) with temperature control (20ºC) using plate-plate serrated geometry with 20 mm diameter and a gap of 0.35 mm. Frequency sweeps were performed in the frequency range of 0.1 - 10 Hz, at a constant strain within the linear region of viscoelastic region of each material, where no variation of G' and G'' is observed (5 Pa for the [BMIM][Cl]-based ionomaterial and hybrid materials, 50 Pa for the [BMIM][DCA]-based ionomaterial, 20 Pa for the [BMIM][DCA]-based hybrid material and 50 Pa for the gelatin hydrogel)

Ionic conductivity measurements

For the characterization of the materials' conductivity properties, 100µl of [BMIM][Cl]-based gelatin ionomaterial were pipetted into a 0.5 ml eppendorf tube previously filled with polydimethylsiloxane (PDMS). Gold coated connection pines, used as electrodes, were inserted in an in-house 3D printed adapter and position in the Eppendorf tube being partially submersed in the material. Likewise, [BMIM][DCA]-based gelatin ionomaterials were prepared using in-house 3D printed o-rings with 6 mm inner diameter and 0.6 mm height. Before the measurement, the ionomaterial was sandwich between two gold electrodes.

Ionic conductivity of the ionomaterials was determined at room conditions by Electrochemical Impedance Spectroscopy using a potentiostat Gamry Instruments-Reference 3000 (measurement conditions: frequency range from 100kHz – 0.1 Hz and V 100mV/ms (AC)). The conductivity was calculated using the following equation

$$\sigma = \frac{1}{R}(\frac{l}{A})$$

where $\sigma$is the conductivity, R is the resistance; and l and A are the thickness and area of the samples, respectively. Experimental data was acquired in duplicates.

Atomic Force Microscopy (AFM)

Atomic force microscopy measurements were conducted in an Asylum Research MFP-3D Standalone AFM system operated in alternate contact mode using commercially available silicon probes (Olympus AC240TS) with a resonance frequency of 70 kHz and a spring constant of 2 Nm^{-1}. Images were processed with Gwyddion software after being plane-fitted/leveled.

Observation of the optical response of hybrid films to humidity by Polarizing Optical Microscopy (POM)

Thin films of each hybrid material formulation were observed in transmission mode between crossed (90°) and semi crossed polarizers (45°) and in bright field (BF) using a polarized optical microscope (Zeiss Axio Observer.Z1), equipped with an Axiocam 503 color camera.

To visualize the optical response of a hybrid film to varying relative humidity (RH), the film was placed inside a custom-made hermetic and transparent chamber, to which a nitrogen stream with known flow rate carrying 80% or 0% RH was alternately injected, and observed in real-time under POM with crossed and semi-crossed polarizers. The changes in molecular ordering of the liquid crystal and alterations in the matrix during exposure to humid and dry nitrogen gas were video recorded using the microscope camera and software (Zeiss ZENPro 2.6). ImageJ software (National Institutes of Health, USA) was then employed to split the videos in frames and calculate the mean grey value for each frame. The relative light intensity transmitted through the film (the film brightness) during the experiment was calculated by normalizing the mean grey value of each frame to the maximum observed grey value and plotted against time.

Optical and electrical signal acquisition devices with measurement of relative humidity (RH) in the detection chamber

The setup employed to record electrical and optical signals generated by ionomaterial and hybrid thin films upon exposure to varying humidity and VOCs is depicted in Figures S3 and S4, respectively.

An optical signal acquisition device assembled in-house, and described in previous publications [1,3,4], was used for measuring the optical response of hybrid films to gas samples.

Briefly, the optical signal acquisition system converts the intensity of light transmitted through the films to voltage. The hybrid material films, spread over transparent glass slides, are placed between crossed polarizers in the detection chamber, which is closed hermetically. As polarized light pass through the films, the radial liquid crystal droplets alter its polarization state thus allowing it to pass through the second polarizer and to reach a photodiode (Figure S18a), which yields a voltage signal. When exposed to a VOC sample, the liquid crystal ordering is disturbed until the liquid crystal becomes isotropic. In this situation, the medium is no longer birefringent and, as such, no light reaches the photodiode (Figure S18b), which results in the photodiode returning the maximum voltage signal. The alternation of liquid crystal ordering between radial configuration and isotropic (Figure S18c) generates a typical waveform response (Figure. S18d) that allows to identify the VOC sample [1,3,4]. The optical responses of hybrid films to nitrogen gas with variable RH were acquired using this device.

The electrical signal acquisition device was also assembled in-house and is described in a previous publication[4]. Briefly, the device measures the electrical conductance of the films, which is presented as voltage with variable gain to better match the analog reading window of the Arduino due microcontroller device that records the signals. Conductance can be measured by relating this output voltage with the variable gain resistor value in the current-to-voltage module [5]. The ionomaterial films, spread over interdigitated gold electrodes imprinted on glass substrates, are placed in the sensor slots of the detection chamber, which is closed hermetically. A 100 mV peak-to-peak 2 kHz triangle wave AC voltage is applied to the interdigitated electrodes. The conductivity meter [5] than measures the variations of conductance of the ionomaterials during exposure to gas samples. Prior to each experiment, the variable resistor in the current-to-voltage module is manually adjusted so that each sensor's baseline (i.e. voltage output signal) is levelled near the minimum of the Arduino microcontroller analog reading window.

Humidity reference sensors (HTU21D-F, Adafruit, New York, USA, with accuracy of ± 2% RH and response time between 5 - 10 s at 63% of the signal) were installed in the supersaturated salt solution vial to record the generated RH (Figure S3) and at the outlet of the detection chamber of the signal acquisition devices to record the variations of relative humidity in the chamber (Figure S4). The measurements of the RH sensor in the supersaturated salt solution vial were synchronized with the measurement of the optical or electrical signal using an Arduino UNO that is synchronized with the signal acquisition device. The measurements of the outlet RH sensor were acquired using an independent Arduino DUE, that also controls the signal acquisition device.

Optical and electrical signal acquisition of ionomaterials and hybrid materials upon exposure toto controlled RH variations

To generate controlled levels of relative humidity, the sample delivery system depicted in Figure S3 was assembled and adapted to the optical or to the electrical signal acquisition device. Two mass flow controllers (MFC1, MC-5SLPM-D/5M, Alicat Scientific Inc; and MFC2, MC-2SLPM-D/5M, Alicat Scientific Inc.) were fed with nitrogen as a carrier gas. A glass vial containing a supersaturated salt solution at room temperature (22 ± 2 °C) was installed at the outlet of the MFC2. Distinct RH levels were generated in the detection chambers by bubbling

the nitrogen stream in supersaturated solutions of different salts [6] (Table S1) before entering the detection chamber.

Table S1. Supersaturated inorganic salt solutions and corresponding maximum relative humidity (RH) levels measured the at ~20º C at the outlet of the detection chamber. Distilled water generates the maximum relative humidity.

Supersaturated salt solution	Maximum RH at outlet of the electrical detection chamber (%)	Maximum RH at outlet of the optical detection chamber (%)
**Magnesium Chloride**	29	25
**Potassium Carbonate**	Not used	36
**Sodium Bromide**	49	50
**Sodium Chloride**	58	65
**Distilled Water**	68	80

The sensing films (ionomaterials or hybrid materials containing either [BMIM][DCA] or [BMIM][Cl]) were placed in the detection chamber of the signal acquisition device and exposed to five humidification-drying cycles, for each RH level (Table S1). To establish the humidification-drying cycles, MFC1 flow rate was constant at 1.5 slpm and MFC2 was programmed to alternately switch the flow rate between 0 and 1.5 slpm:

- 140 seconds at 1.5 slpm: Humidification period
- 140 seconds at 0 slpm: Drying period

During the humidification period, the films were exposed to humidified nitrogen. During the drying period, dry nitrogen purged the detection chamber to ensure 0% RH (humidification and drying courses in Figure S4).

For the experiments with ionomaterials, the sensors were previously equilibrated at 0% RH for 15 minutes, thus the first cycle started from 0% RH. For the experiments with hybrid materials, there was not an equilibration period, therefore the first cycle started in variable RH (room RH).

The sensors were stored under controlled humidity (around 50 – 60% RH) during a maximum period of 1 week before being used in the experiments. We used three independent sensors to test each RH level.

Electrical and optical signal processing and features extraction

The raw signals yielded by the ionomaterials (electrical signal) and hybrid materials (optical signal) were firstly smoothed with a hanning window of 50 points and filtered with a median filter of variable size to remove unwanted noise. Then, using a custom-made Python 3.7 script, the resulting signals were divided in cycles (in general, 5 cycles of exposure/recovery to VOC or RH were performed) and each individual cycle was centered in zero and normalized to its baseline, to yield the *Relative Signal*, according to equation 4:

$$Relative\ signal\ (Rs) = \frac{signal - baseline}{baseline} \qquad \text{(equation 4)}$$

Where *signal* is the smoothed and filtered individual cycle signal, and *baseline* is the average of the *signal* immediately before (10 sample points) the exposure period starts.

This methodology was applied to calculate both the relative electrical signal and the relative optical signal.

It is important to analyse the results in the form of relative signals because there is a certain degree of variability of the baseline of the electrical and optical signal between different sensors. By analysing the relative signals, the effect of this variability is removed and thus, it is possible to compare the relative response of different sensors.

Then, the following features were extracted: *response time*, defined as the time needed for the relative signal to reach 90% of its maximum variation; *recovery time*, defined as the time needed for the relative signal to recover until 10% of its maximum variation after the exposure period stops; and the *relative sensor response* (electrical – Re_r – or optical – Ro_r), defined as the maximum variation of the relative signal (it can be either a maximum or a minimum, depending on the signal shape).

When applicable, the sensors' VOC responsivity rates were determined as the slope of the linear function that better fits the profile of sensor relative response variation in function of the VOC concentration.

Determination of mass variation of hybrid [BMIM][DCA] and [BMIM][Cl] hybrid material films upon exposure to controlled variations of RH

Triplicates of hybrid films were stored in a controlled humidity chamber (RH = 50 ± 5%) previously to the assay. Then, using the setup depicted in Figure S3, the films were exposed to RH variations in the sequence: 50% → 0% → 85% → 0%. The mass of the films was measured immediately before the assay and after each RH variation period. The relative mass variation was determined according to equation 1

$$\Delta mass\ (\%) = 100\ (\%) \times \frac{RH_{final}\ (mg) - RH_{initial}\ (mg)}{RH_{initial}\ (mg)} \qquad \text{(equation. 1)}$$

where $RH_{initial}$ and RH_{final} are the mass of the thin films at the beginning and end of each period of exposure to nitrogen gas, respectively.

Generation and sampling of different volatile organic compound (VOC) concentrations under controlled RH

To sample known concentrations of VOC (ethanol, acetone, toluene and hexane) under controlled levels of RH to the detection chamber of the signal acquisition device, the setup depicted in Figure S4 was assembled. The dilution MFC (MC-5SLPM-D/5M, Alicat Scientific Inc.) was fed with nitrogen gas, which was bubbled through NaBr to generate a RH of 50% in the detection chamber, or sent directly to the detection chamber to ensure a RH of 0%. The carrier MFC (MC-2SLPM-D/5M, Alicat Scientific Inc.) was also fed with nitrogen gas, which was bubbled through a known volume of pure solvent to generate a known flow rate of VOC vapor in the nitrogen carrier stream.

The bubbler system is composed by a dip tube attached to a porous filter. The flow rate of VOC vapor and water vapor at the output of the bubbler flask, *Fn*, was calculated using the bubbler equation (equation 2) [7–9]

$$F_n = \frac{P_n^*}{P_i} \times F_c, \qquad n = v\ (VOC);\ w\ (water) \qquad \text{(equation. 2)}$$

where P^*_n is the saturated vapor pressure of the solvent or of water at a given temperature, calculated by the Antoine Equation, or corresponding to the registered RH, respectively; P_i is the pressure of the inlet stream, given by the carrier MFC; and F_c is the flow rate of the nitrogen carrier stream.

Different VOC concentrations in the detection chamber were generated by varying the temperature of the solvent (20º - 28º C) and by mixing the carrier flow rates (nitrogen flow rate (F_c) and VOC flow rate (F_v)) generated at the output of the bubbler flask with the dilution flow rates (nitrogen flow rate (F_d) and water vapor flow rate, if present (F_w)) at different ratios. The resulting VOC concentration, in *%(v/v)*, (Table S2) were calculated from F_v and the total mixed flow rates (equation 3).

$$C_{VOC}\ (\%\ (v/v)) = \frac{F_v \times 10^6}{F_c + F_v + F_d + F_w}\ (ppm) \times 10^{-4} \left(\frac{\%\ (v/v)}{ppm}\right) \qquad \text{(equation 3)}$$

The dilution MFC was set at a fixed flow rate (1.5 slpm, for ethanol, toluene and hexane or 5.0 slpm, for acetone). The carrier MFC was programmed to vary the flow rate in increments of 0.05 slpm (between 0.05 and 1.5 slpm) and repeat five times each flow rate step. In this way, five replicates of each VOC concentration were sampled to the detection chamber of the signal acquisition device during exposure/recovery cycles.

To generate the exposure-recovery cycles, a two-way solenoid valve was used. The valve was programmed to alternately direct the nitrogen carrier stream (Figure S4) to the exposure or the recovery path using an automated temporized switching:

- 5 seconds ON – Exposure
- 15 seconds OFF – Recovery

During the exposure period, the nitrogen carrier stream bubbles through the solvent, is mixed with the dilution nitrogen stream (dry or humid) and, finally, introduced in the detection chamber. During the recovery period, the carrier stream is directed to an exhaust line and the detection chamber is purged with dry or humid nitrogen gas.

A custom made python script (Python 3.7, using alicat library 0.2.2) was used to program the carrier MFC and solenoid valve operation, as well as to synchronize them with the signal acquisition device and the readings of the reference RH sensor at the outlet of the detection chamber.

Table S2. Carrier (Fc) and dilution (Fd) flow rates and corresponding VOC concentrations to which the [BMIM][DCA] and [BMIM][Cl] ionomaterial and hybrid material films were exposed.

	Fd = 5 slpm		Fd = 1.5 slpm	
**Fc (slpm)**	**Acetone (% v/v)**	**Ethanol (% v/v)**	**Toluene (% v/v)**	**Hexane (%v/v)**
0.05	0.30	0.30	0.12	0.63
0.1	0.59	0.49	0.23	1.22
0.15	0.88	0.71	0.34	1.76
0.2	1.16	0.92	0.44	2.27
0.25	1.43	1.11	0.53	2.74
0.3	1.69	1.29	0.62	3.19
0.35	1.95	1.46	0.70	3.60
0.4	2.20	1.63	0.78	3.99
0.45	2.45	1.78	0.86	4.36
0.5	2.69	1.92	0.93	4.70
0.55	2.93	2.06	0.99	5.03
0.6	3.16	2.19	1.06	5.34
0.65	3.38	2.32	1.12	5.63
0.7	3.60	2.44	1.18	5.91
0.75	3.82	2.55	1.23	6.17
0.8	4.03	2.66	1.28	6.43
0.85	4.23	2.76	1.33	6.66
0.9	4.44	2.86	1.38	6.89
0.95	4.63	2.95	1.43	7.11
1	4.83	3.04	1.47	7.32
1.05	5.02	3.13	1.52	7.52
1.1	5.20	3.21	1.56	7.71
1.15	5.38	3.29	1.60	7.89
1.2	5.56	3.37	1.63	8.07
1.25	5.74	3.45	1.67	8.23
1.3	5.91	3.52	1.71	8.40
1.35	6.08	3.59	1.74	8.55
1.4	6.24	3.65	1.77	8.70
1.45	6.40	3.72	1.80	8.84

The limit of detection (LOD) was considered as the minimum VOC concentration that triggered an increase in the amplitude of the ionomaterial or hybrid sensor signal relative to the baseline. The saturation state for each VOC was determined as the concentration above which there was no increase in the amplitude of the sensors optical or electrical signals to the VOC.

Measurement of the optical and electrical signals of [BMIM][DCA] and [BMIM][Cl] ionomaterials and hybrid materials sensing films upon exposure to different concentrations of VOCs under controlled RH

Duplicates or triplicates of films made of ionomaterial or hybrid material (containing either [BMIM][DCA] or [BMIM][Cl]) were placed in the electrical or optical detection chamber, respectively, and submitted to a total of 145 exposure-recovery cycles to each VOC (ethanol, acetone, toluene and hexane), five cycles at each concentration, under two distinct RH conditions (0% and 50% RH).

Prior to each experiment, the signals were allowed to stabilize under the test %RH, by purging the detection chamber with dry or humid nitrogen for 15 minutes. To observe the slight changes in RH due to the added carrier flow rate during exposure on the electrical and optical response of the ionomaterial and hybrid material films, blank assays without VOCs were conducted for each tested ratio of carrier and dilution flow rates.

Supplementary Figures and Tables

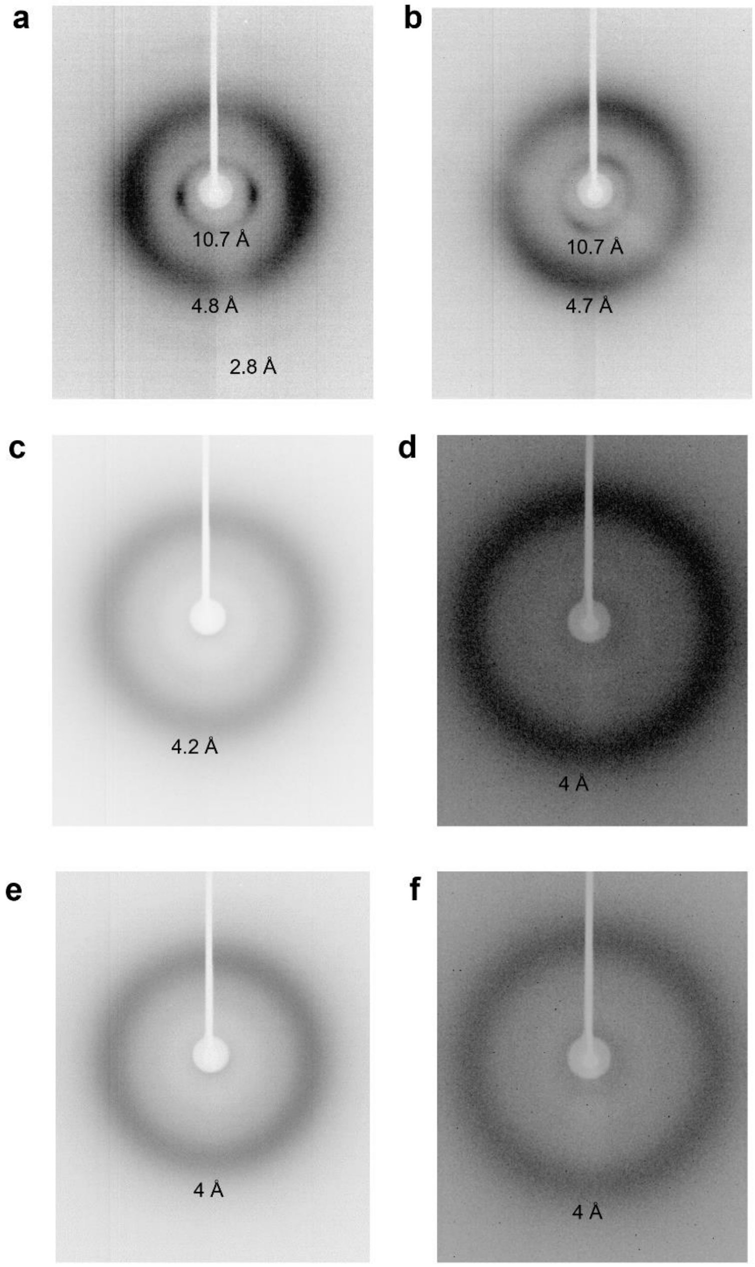

Figure S1. X-ray scattering images of the gelatin-based materials. a) hydrogel composed by gelatin and water. **b)** control material composed of gelatin and 5CB. **c)** ionomaterial composed by gelatin, [BMIM][DCA] and water. **d)** ionomaterial composed by gelatin, [BMIM][Cl] and water. **e)** hybrid material composed by gelatin, [BMIM][DCA], 5CB and water. **f)** hybrid material composed by gelatin, [BMIM][Cl], 5CB and water.

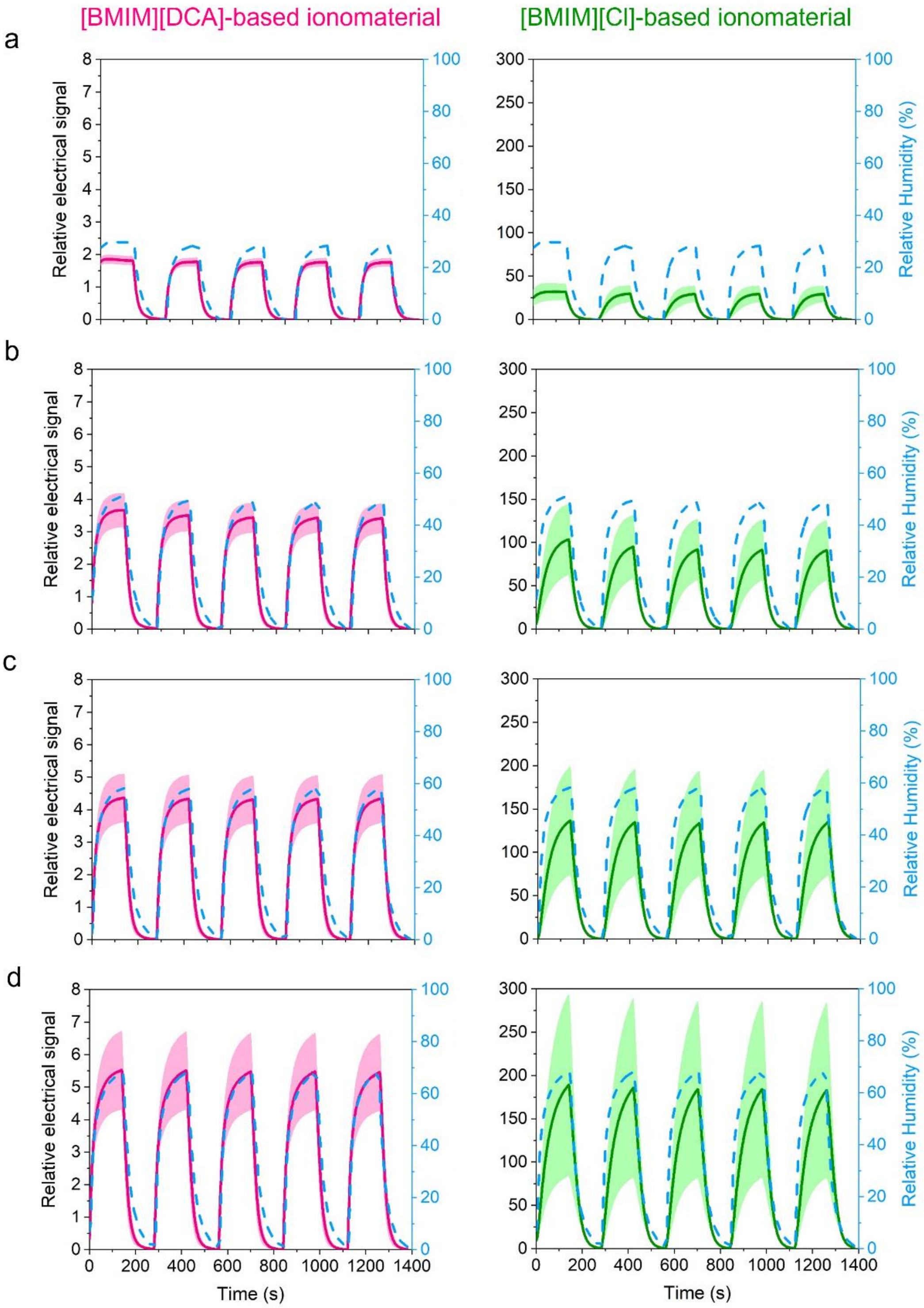

Figure S2. Repeatability of the electrical signal of [BMIM][DCA] and [BMIM][Cl]-based gelatin ionomaterial thin films upon exposure to humidification-drying cycles between 0% and a) 30, b) 50, c) 60 and d) 70 %RH. The full line and shadows represent, respectively, the average signal and standard deviation of three independent sensors.

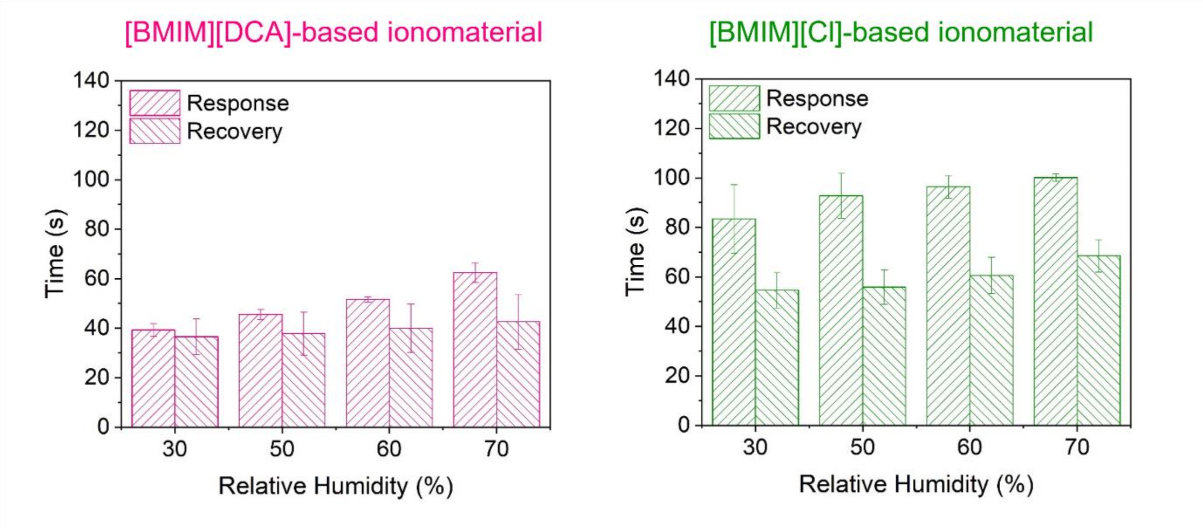

Figure S3. Analysis of the electrical response to humidity of [BMIM][DCA] and [BMIM][Cl] ionomaterial thin films. Response and recovery times to humidity changes from 0% to 25%, 35%, 50%, 60% and 70% (n = *15*).

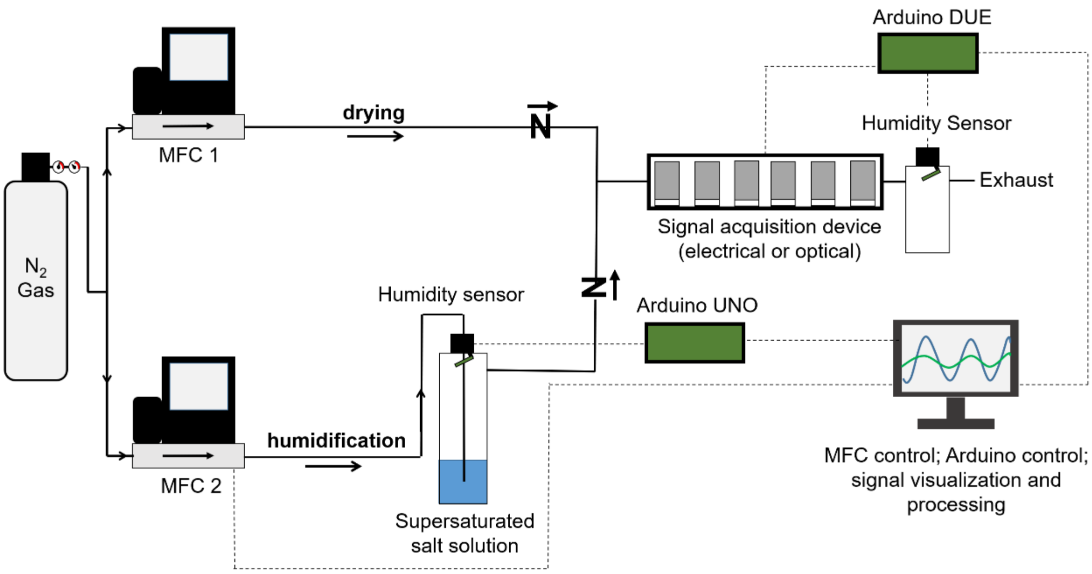

Figure S4. Experimental setup for humidity exposure assays. Controlled relative humidity levels are generated with the gas delivery system composed by two mass flow controllers (MFC) and a bubbling system fed with nitrogen. The generated nitrogen currents are input to the signal acquisition device containing the optical or electrical sensors in such a way that the sensors are alternately exposed to dry and humid nitrogen.

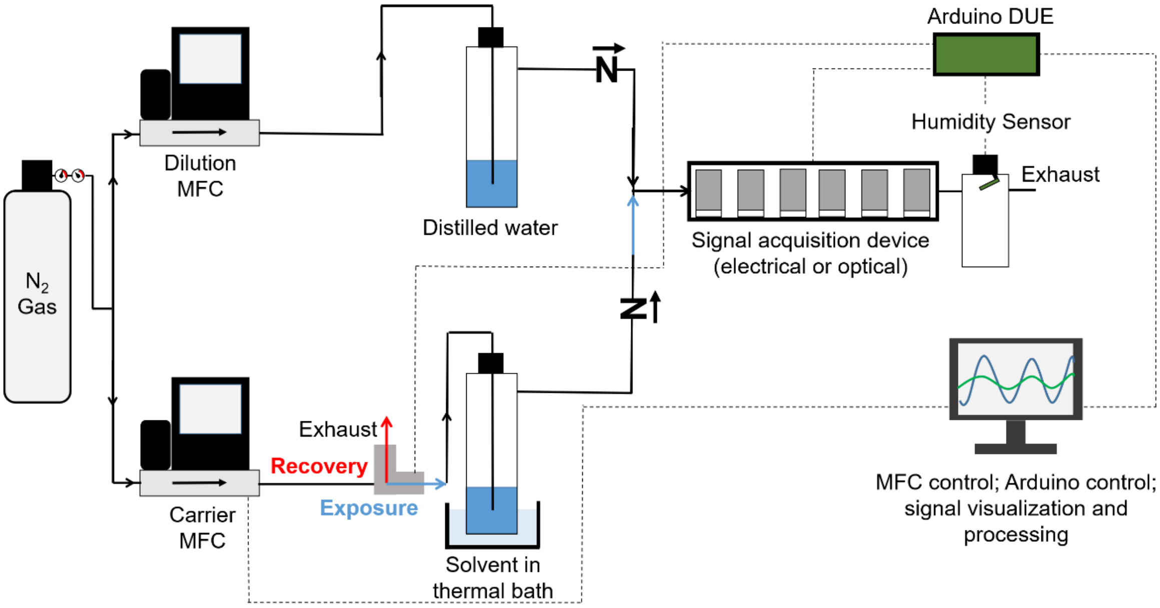

Figure S5. Experimental setup for VOC exposure assays. VOC concentrations in dry or humid nitrogen are generated by manipulating the temperature of the solvent in the thermal bath and the flow rates of the dilution and carrier mass flow controllers (MFC). The working principle of the setup can be described as follows. In the gas exposure periods, the nitrogen carrier stream (flow rate controlled by the carrier MFC) is bubbled through the liquid solvent to vaporize it and mix with the VOC. The dilution nitrogen stream (flow rate controlled by the dilution MFC) can be mixed directly with the carrier stream (for 0% RH) or first bubbled through water (for 50% RH). The carrier and dilution stream mix at the inlet of the sensors chamber, thus generating the exposure stream. In the recovery periods, only the dilution stream enters in the sensors chamber.

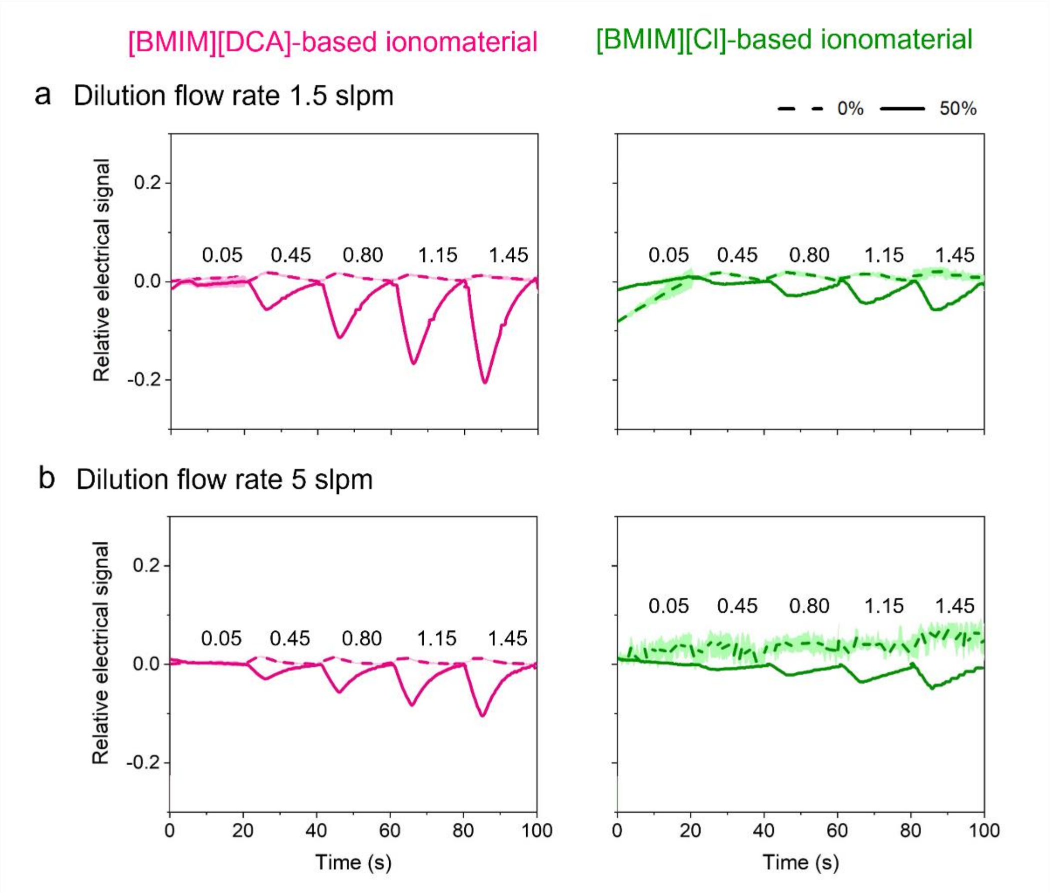

Figure S6. Relative electrical signal generated by [BMIM][DCA] and [BMIM][Cl] ionomaterial thin films in the blank assay, upon exposure to dry (0% RH, dashed line) and humid (50% RH, full line) nitrogen in the absence of VOCs, at dilution flow rates of a) 1.5 slpm and b) 5.0 slpm, which were used afterwards to manipulate VOC concentration. The responses to 5 carrier flow rates, between 0.1 and 1.45 slpm, are represented for each dilution flow rate. The lines and shadows represent the average and standard deviation of 5 measurements (*n=5*).

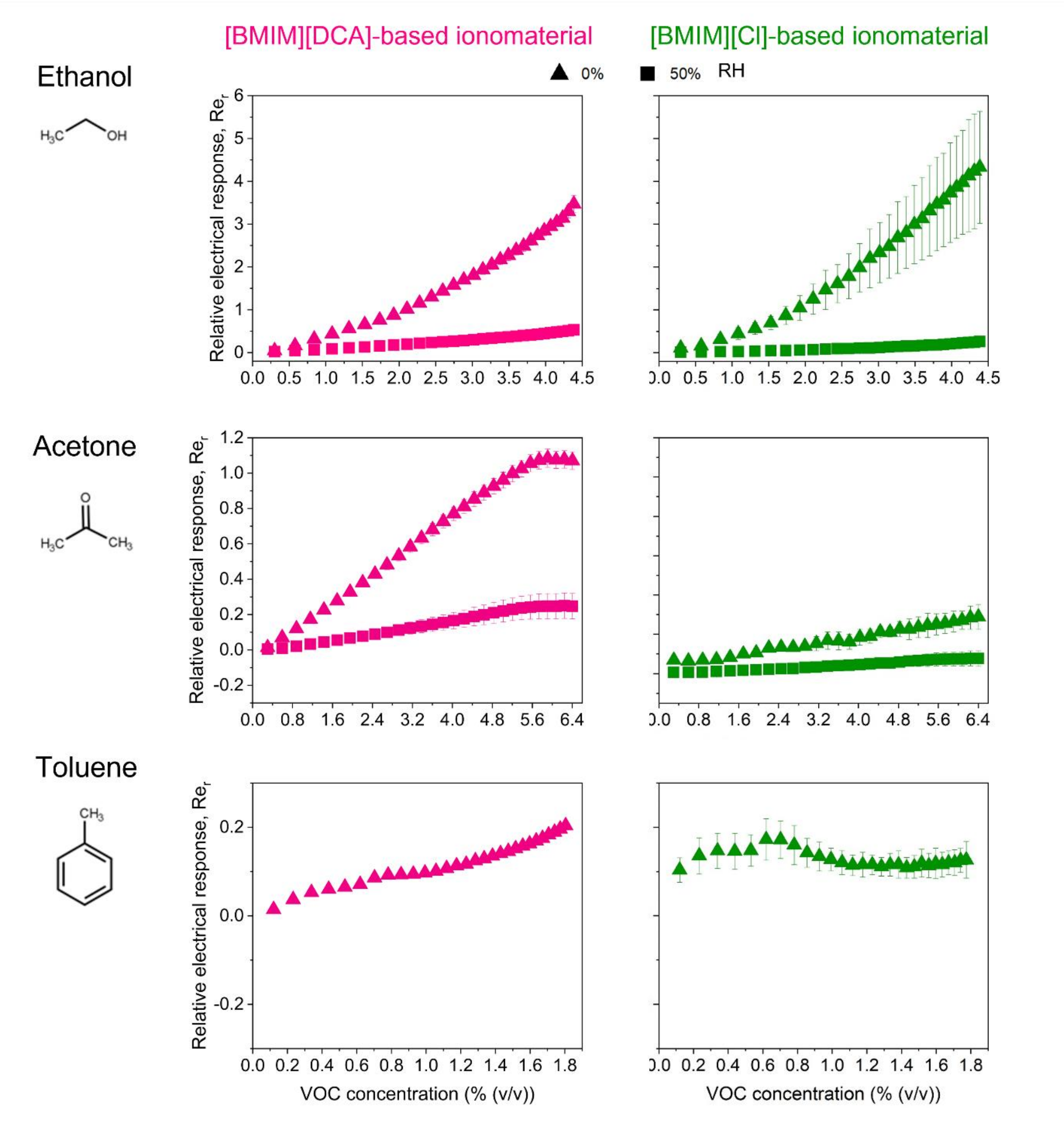

Figure S7. **[BMIM][DCA] and [BMIM][Cl] ionomaterial sensors relative electrical response when exposed of ethanol, acetone and toluene under dried and humidifed condictions.** (*n=5*).

Table S3. VOC sensing electrical performance of [BMIM][DCA]-based materialatin ionomaterial films under dry (0% RH) and humidified environments (50% RH).

VOC	0% RH<br>LOD<br>(%(v/v))	 Resp. rate<br>(V/%(v/v))	 Sat. conc.<br>(%(v/v))	50% RH<br>LOD<br>(%(v/v))	 Resp. rate<br>(V/%(v/v))	 Sat. conc.<br>(%(v/v))
Ethanol	nd	exponential	nd	nd	0.08	nd
Acetone	nd	0.20	5.7	0.1	0.03	5.4
Toluene	nd	llinear	nd	-	-	-

VOC, Volatile organic compound; LOD, Limit of detection ; Resp. rate, Responsivity rate ; Sat. conc. Saturation concentration ; nd, not detected; -, nor applicabe (no quantitative response.

Table S4. VOC sensing electrical performance of [BMIM][Cl]-based gelatin ionomaterial films under dry (0% RH) and humidified environments (50% RH).

VOC	0% RH<br>LOD<br>(%(v/v))	 Resp. rate<br>(V/%(v/v))	 Sat. conc.<br>(%(v/v))	50% RH<br>LOD<br>(%(v/v))	 Resp. rate<br>(V/%(v/v))	 Sat. conc.<br>(%(v/v))
Ethanol	0.6	exponential	nd	nd	0.04	nd
Acetone	nd	0.20	5.6	nd	linear	nd
Toluene	nd	nd	0.1	-	-	-

VOC, Volatile organic compound; LOD, Limit of detection ; Resp. rate, Responsivity rate ; Sat. conc. Saturation concentration ; nd, not detected; -, nor applicabe (no quantitative response.

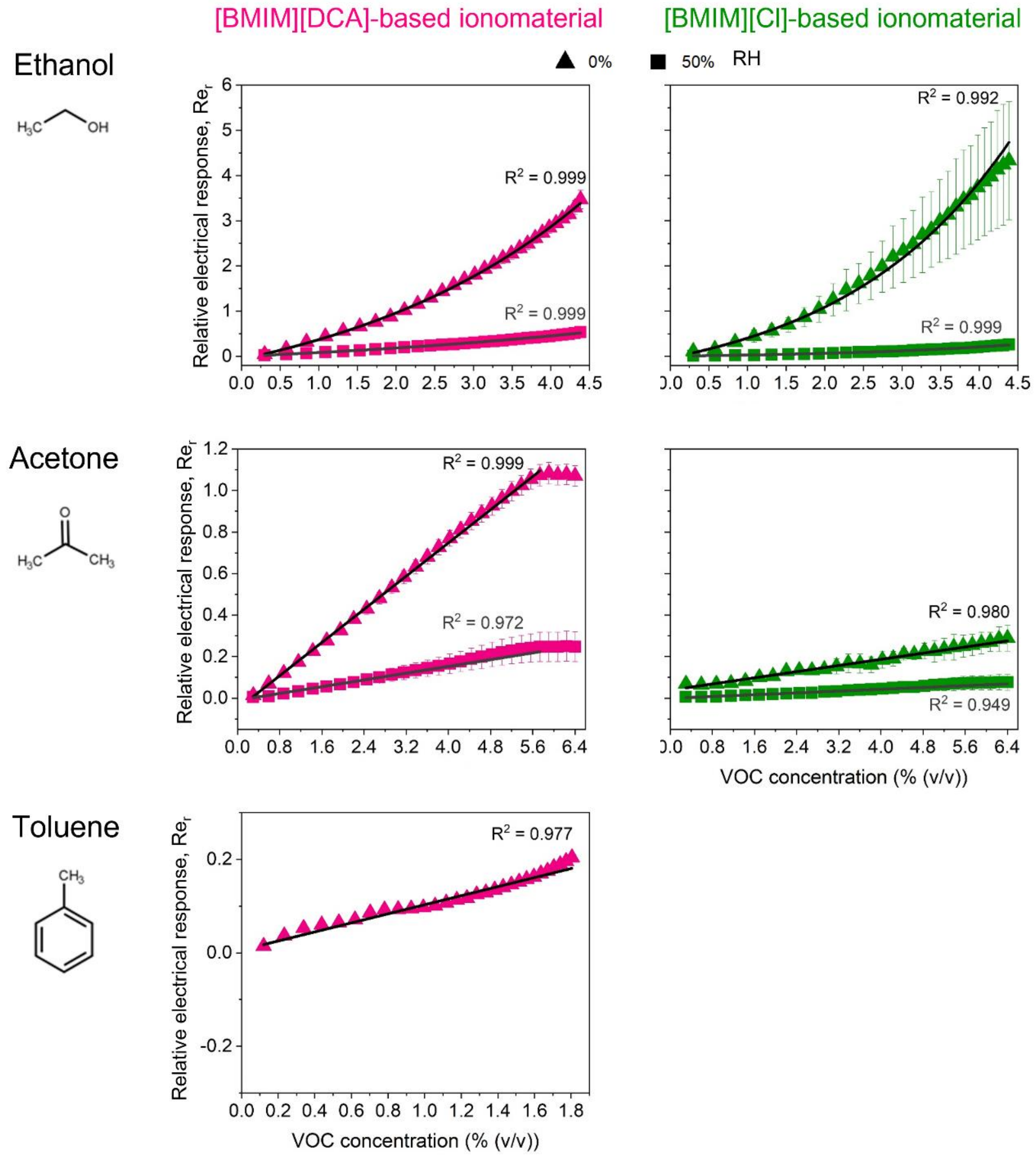

Figure S8. Fitting of the electrical relative response (Re_r) profile of [BMIM][DCA] and [BMIM][Cl] ionomaterial thin films to ethanol, acetone and toluene at 0% and 50% RH.

Ethanol sensing (exponential fitting):

For [BMIM][DCA] ionomaterials at 0%: $rRe_r = -1.264 + 1.310\, e^{\left(\frac{VOC_{conc} - 0.287}{3.234}\right)}$

For [BMIM][DCA] ionomaterials at 50%: $Re_r = -0.386 + 0.386\, e^{\left(\frac{VOC_{conc}}{5.169}\right)}$

For [BMIM][Cl] ionomaterials at 0%: $Re_r = -0.796 + 1.067\, e^{\left(\frac{VOC_{conc} - 0.0.742}{2.214}\right)}$

For [BMIM][Cl] ionomaterials at 50%: $Re_r = -0.042 + 0.046\, e^{\left(\frac{VOC_{conc} - 0.029}{2.358}\right)}$

Acetone sensing (linear fitting):

For [BMIM][DCA] ionomaterials at 0%: $Re_r = -0.052 + 0.200\ VOC_{conc}$

For [BMIM][DCA] ionomaterials at 50%: $Re_r e = -0.010 + 0.041\ VOC_{conc}$

For [BMIM][Cl] ionomaterials at 0%: $Re_r = 0.038 + 0.037\ VOC_{conc}$

For [BMIM][Cl] ionomaterials at 50%: $Re_r = -0.001 + 0.011\ VOC_{conc}$

Toluene sensing (linear fitting):

For [BMIM][DCA] ionomaterials at 0%: $Re_r = 0.006 + 0.097\ VOC_{conc}$

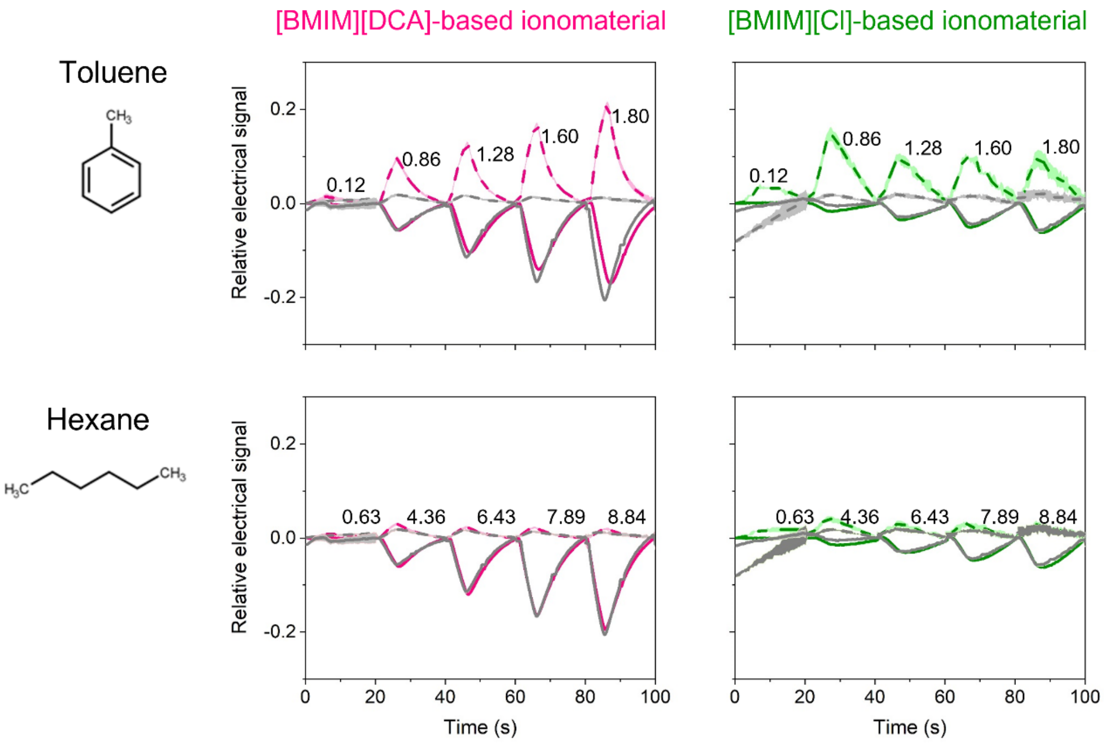

Figure S9. Relative electrical signal of [BMIM][DCA] and [BMIM][Cl] ionomaterial thin films to toluene and hexane diluted in dry (0% RH, dashed line) or humidified (50% RH, full line) nitrogen in comparison with the blank signal. VOC concentrations (% (v/v)) are

indicated on the plots for each condition (*n* = *5*). Signals obtained for the blank assay (same as in Figure S5) are represented in grey.

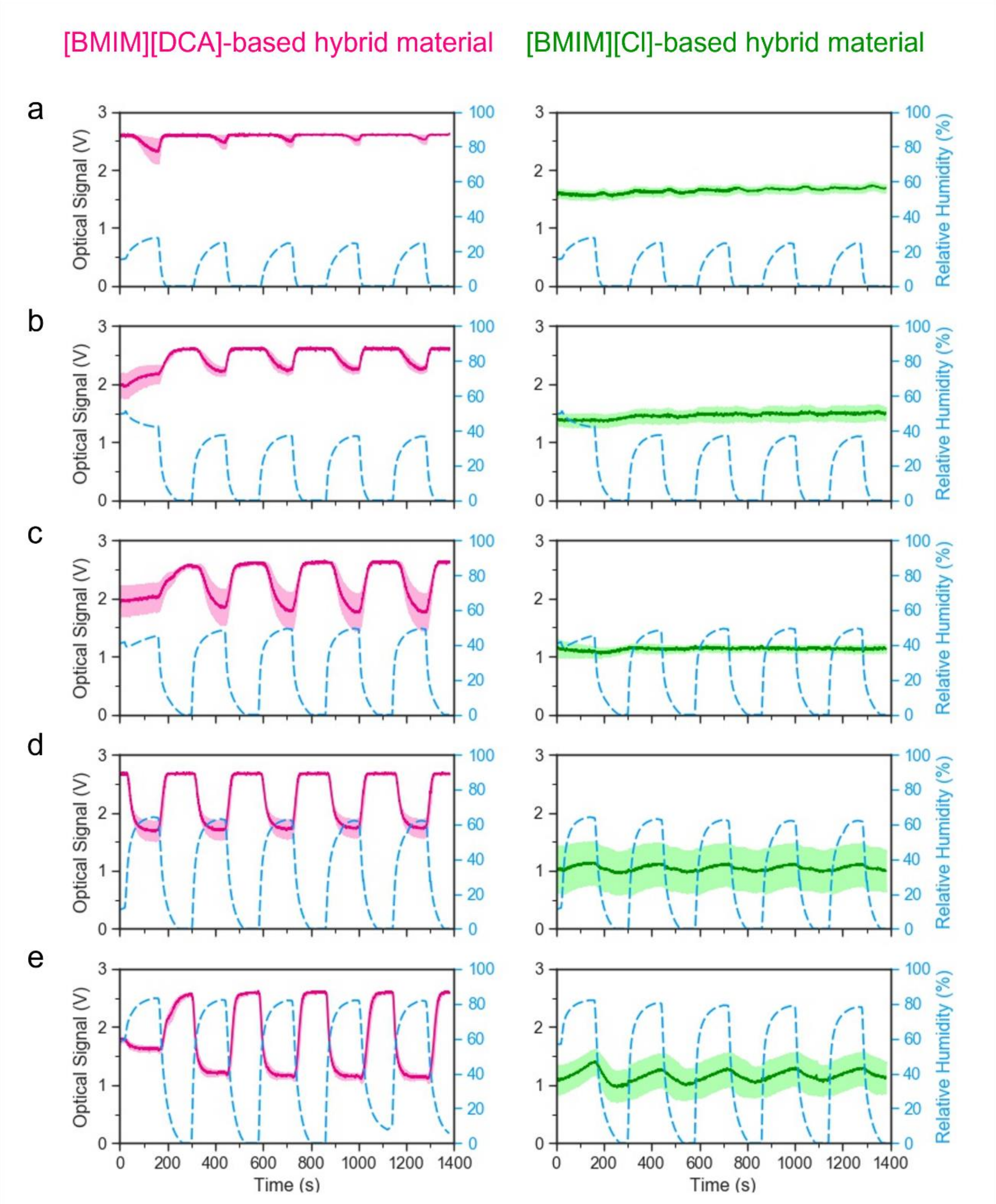

Figure S10. Repeatability of the optical signal of [BMIM][DCA] and [BMIM][Cl] hybrid material thin films upon exposure to humidification-drying cycles between 0% and a) 25, b) 35, c) 50, d) 60 and e) 80 %RH. The full lines and shadows represent, respectively, the average signal and standard deviation of two or three independent sensors.

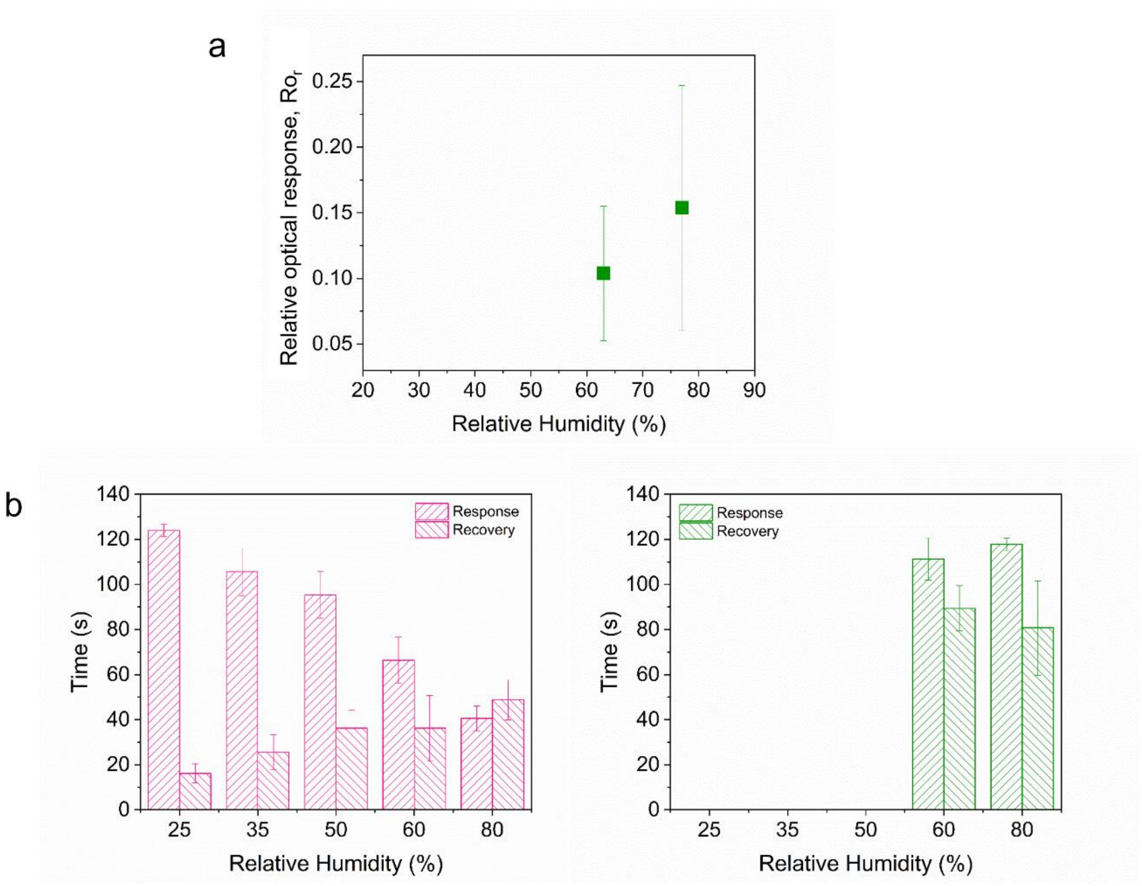

Figure S11 Analysis of the optical response to humidity of [BMIM][DCA] and [BMIM][Cl] hybrid material thin films. a) Variation of the sensors relative optical response (Ro_r) as a function of the RH level (n =12) for [BMIM][Cl] hybrid materials**b)** Response and recovery times to humidity changes from 0% to 25%, 35%, 50%, 60% and 80% (*n = 12 for [BMIM][DCA] and n = 8 for [BMIM][Cl]*). For [BMIM][DCA] films, *response time = -1.484RH + 161.127 (*R^2 *= 0.997*) and *recovery time = 0.600RH + 2.222 (*R^2 *= 0.939).*

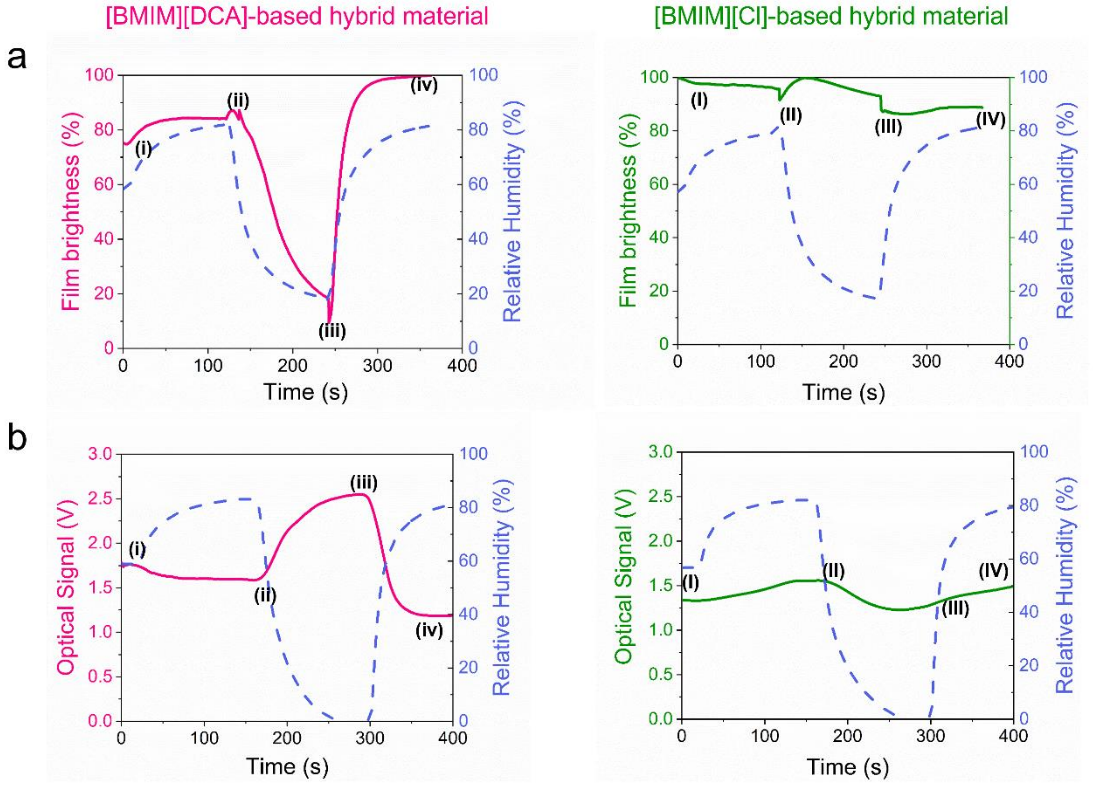

Figure S12. Detail of the optical response to humidity of [BMIM][DCA] and [BMIM][Cl] hybrid material thin films. a) Variation of brightness of hybrid material thin films during exposure to a humid nitrogen stream with the RH profile represented by the blue dashed line. **b)** Optical signal of hybrid material thin films collected in the optical signal transducer during exposure to a humid nitrogen stream with the RH profile represented by the blue dashed line. Points (i – iv and I - IV) correspond to the POM images (i – iv) and (I – IV) in Figure 6 of the main text.

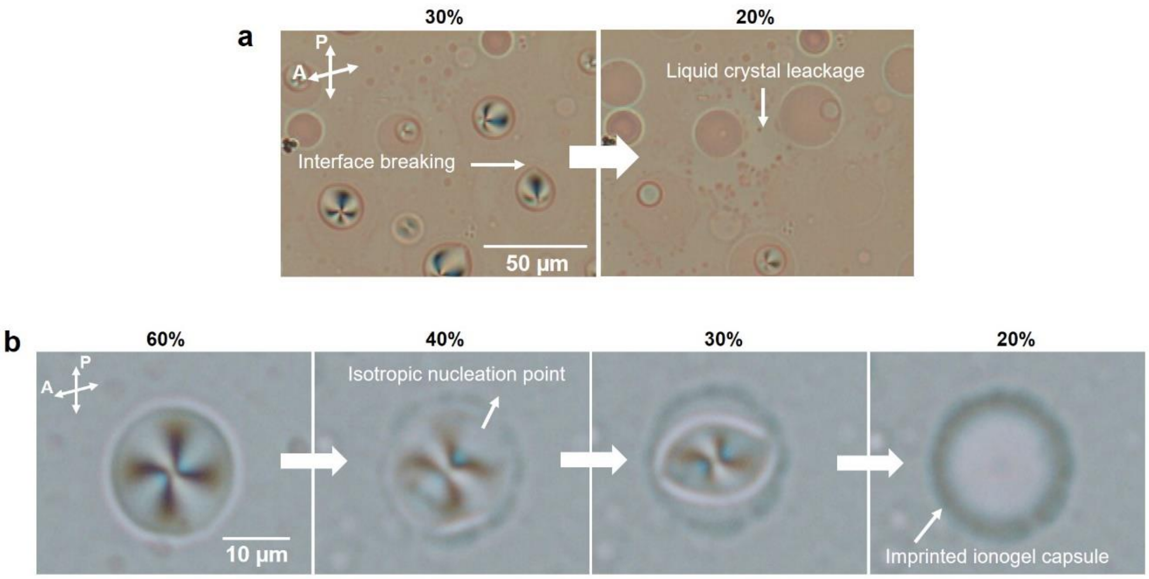

Figure S13. POM images with semi-crossed polarisers revealing details of the morphological changes of a [BMIM][DCA] hybrid material film during drying. a) Shrinking liquid crystal droplets along with liquid crystal ordering transitions and leakage of isotropic liquid crystal to the matrix while the film is dried from 30% RH to 20% RH. **b)** Mechanism of phase transition of the liquid crystal from radial to isotropic when the film is dried 60% RH to 20% RH. The [BMIM][DCA]-stabilized droplet is contained in a capsule imprinted in the matrix and suffers shape and volume changes simultaneous with the formation of nucleation points from where isotropization starts.

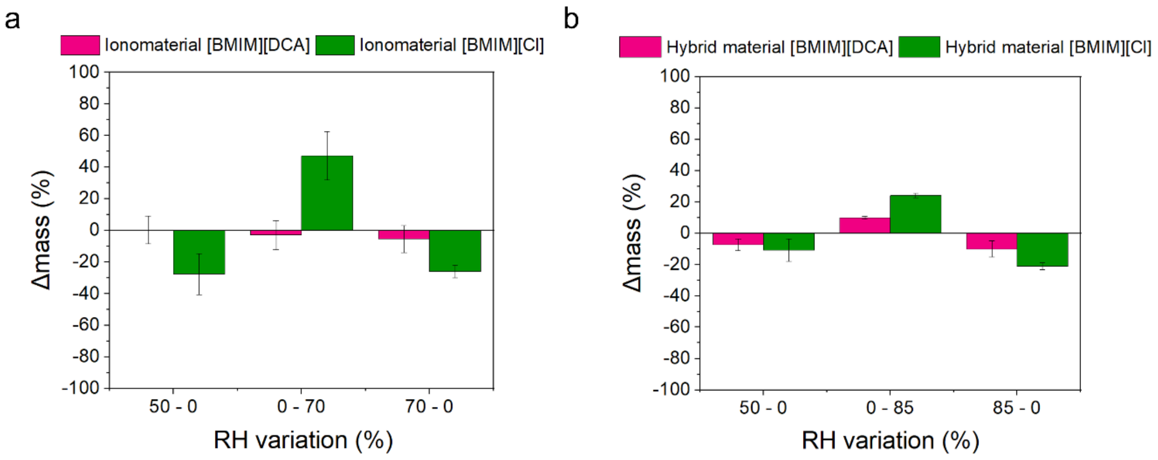

Figure S14. Mass changes of [BMIM][DCA] and [BMIM][Cl] hybrid material films, corresponding to swelling and contraction of the gelatin matrix due to desorption and sorption of water when the films are sequentially exposed to dry, humid and again dry nitrogen current.

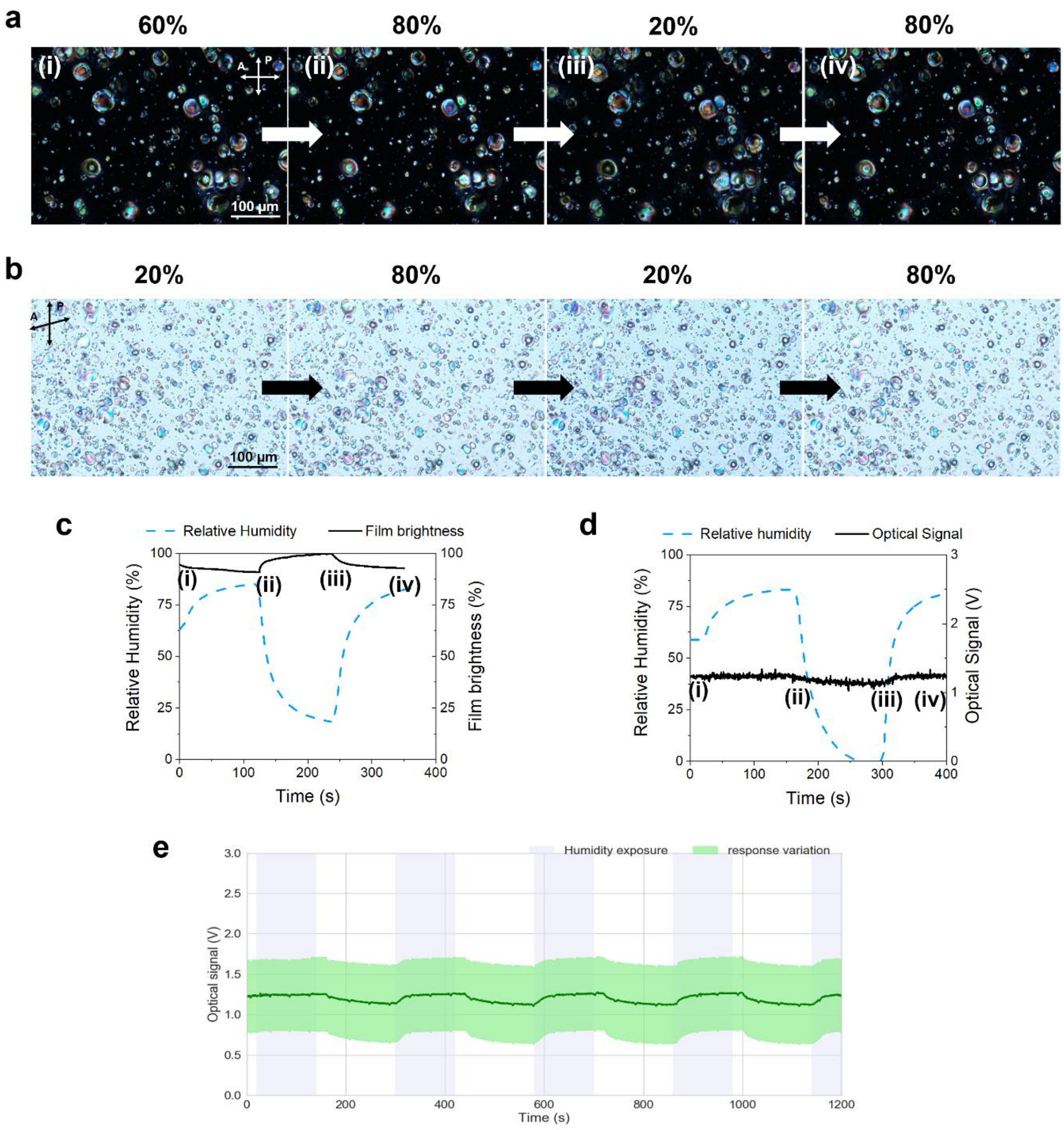

Figure S15. Optical response to humidity of a control material films without ionic liquid. a) Crossed polarizers POM images of a representative area of a control film during sequential exposure to humid nitrogen with relative humidity (RH) varying between (i) 60% (room conditions), (ii) 80%, (iii) 20% and (iv) 80%. b) Semi-crossed polarizers POM images of a representative area of a control film in the same conditions as in (a). c) Variation of brightness of the film in (a) and relative humidity profile to which it was exposed; points (i - iv) in the brightness profile correspond to the POM images (i – iv) in (a). d) Optical signal of a film (acquired with an in-house assembled signal acquisition device) and RH profile to which it was exposed: points (i - iv) in the optical signal correspond to the POM images (i – iv) in (a). e) average optical signal and signal variation (n=3) acquired from a control material with the signal transducer assembled in-house during 4 cycles of exposure to humid (80% RH) and dry (20% RH) nitrogen.

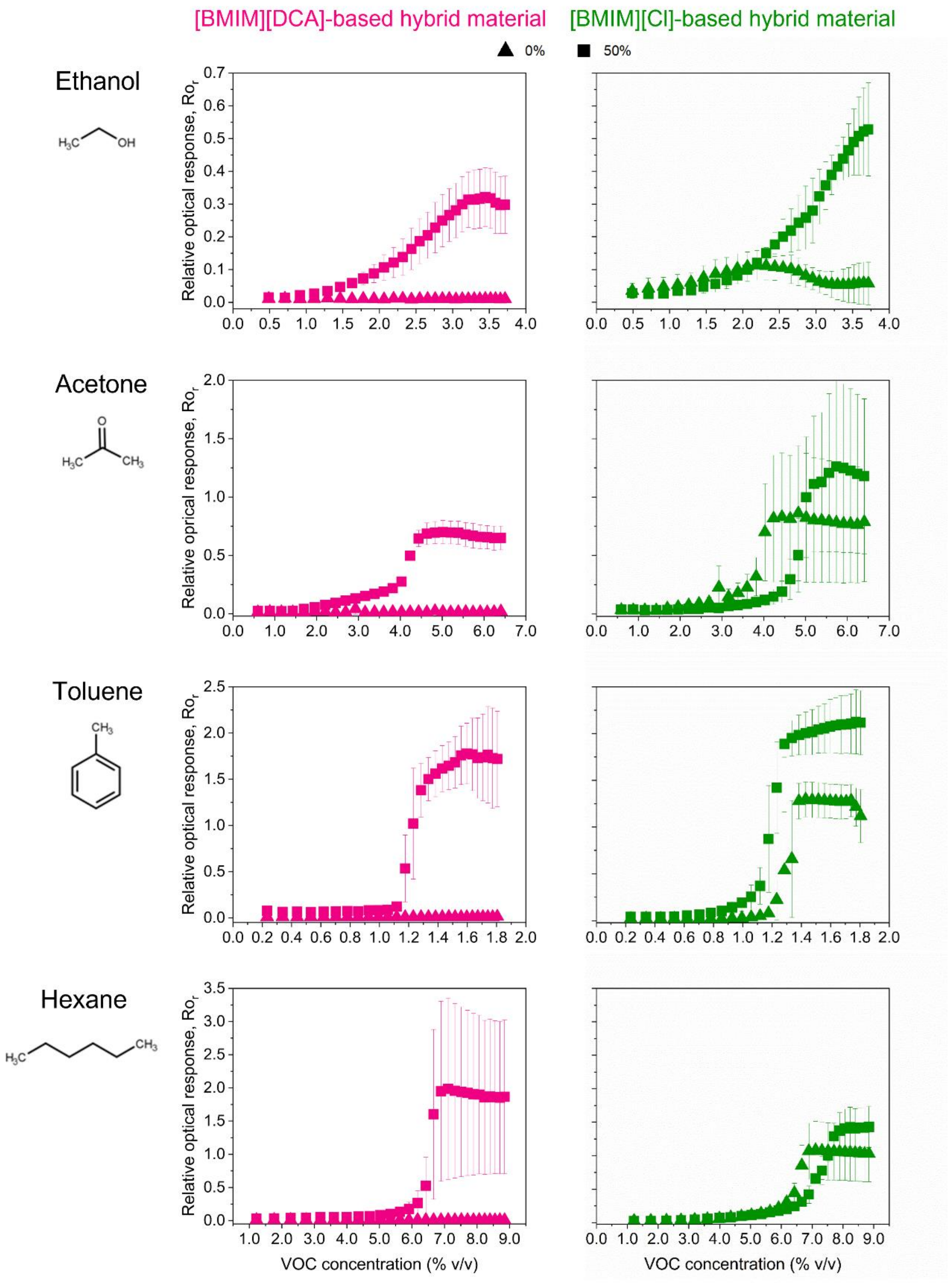

Figure S16. **[BMIM][DCA] and [BMIM][Cl] hybrid material thin films relative optical response when exposed of ethanol, acetone, toluene and hexane under dried and humidifed condictions.** (*n>10*).

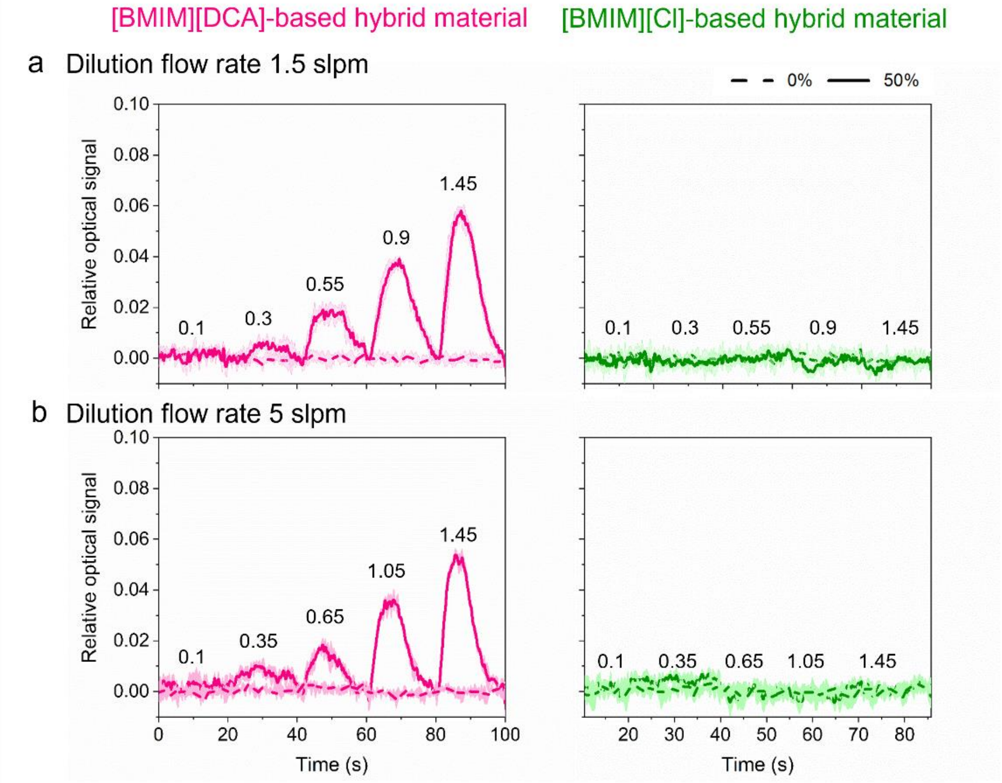

Figure S17. Relative optical signal generated by [BMIM][DCA] and [BMIM][Cl] hybrid thin films upon exposure to dry (0% RH, dashed line) and humid (50% RH, full line) nitrogen in the absence of VOCs, at dilution flow rates of a) 1.5 slpm and b) 5.0 slpm which were used afterwards to manipulate VOC concentration. The responses to 5 carrier flow rates, between 0.1 and 1.45 slpm are represented for each dilution flow rate. The lines and shadows represent the average and standard deviation of 5 measurements (*n=5*).

Table S5. VOC sensing optical performance of [BMIM][DCA] hybrid material thin films under humidified (50% RH) environment.

VOC	LOD (%(v/v))	Triggering conc. (%(v/v))	Sat. conc. (%(v/v))	fitting
Ethanol	1.00	-	3.13	Logistic
Acetone	1.70	4.03	5.02	-
Toluene	1.00	1.06	1.60.	-
Hexane	n.d.	6.17	7.11	-

VOC, Volatile organic compound; LOD, Limit of detection; Sat. conc, Saturation concentration ; nd, not detected; -, not applicable.

Table S6. VOC sensing optical performance of [BMIM][Cl] hybrid material thin films under dried (0% RH) environment.

VOC	LOD (%(v/v))	Triggering conc. (%(v/v))	Sat. conc. (%(v/v))	Fitting
Ethanol	- kj	-	-	-
Acetone	1.70	3.82	4.23	-
Toluene	0.93	1.18	1.43	-
Hexane	2.27	5.90	7.11	-

VOC, Volatile organic compound; LOD, Limit of detection; Sat. conc, Saturation concentration ; nd, not detected; -, not applicable

Table S7. VOC sensing optical performance of [BMIM][Cl] hybrid material thin films under humidified (50% RH) environment.

VOC	LOD (%(v/v))	Triggering conc. (%(v/v))	Sat. conc. (%(v/v))	Fitting
Ethanol	1.10	-	n.d.	Logistic
Acetone	2.20	4.63	5.74	-
Toluene	0.53	1.12	1.77	-
Hexane	2.27	6.17	8.23	-

VOC, Volatile organic compound; LOD, Limit of detection; Sat. conc, Saturation concentration ; nd, not detected; -, not applicable.

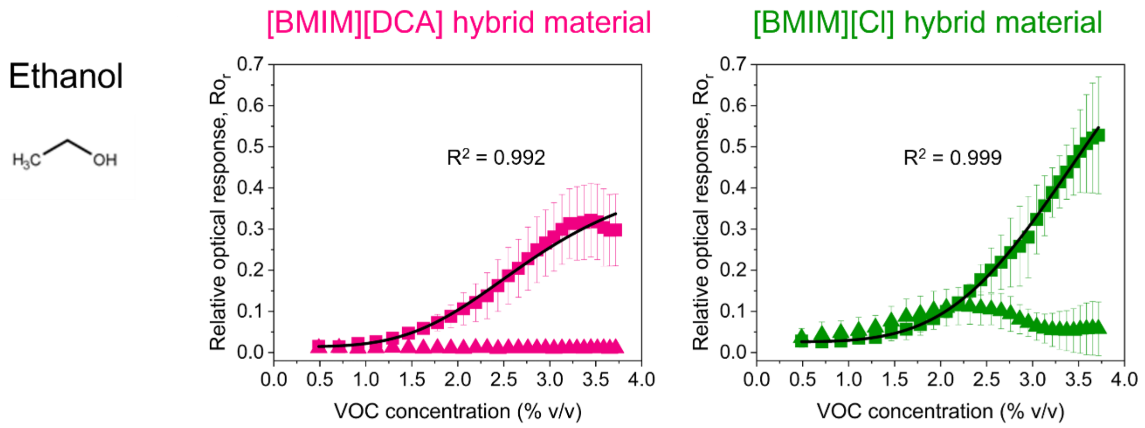

Figure S18. Logistic fitting of the relative optical response (Ro_r) profile of [BMIM][DCA] and [BMIM][Cl] hybrid material thin films to ethanol at 50% RH.

For [BMIM][DCA] hybrid materials: $Ro_r = 0.455 + \frac{0.469}{1+(\frac{VOC\ conc}{2.859})^{3.844}}$.

For [BMIM][Cl] hybrid materials: $Ro_r = 1.137 + \frac{1.163}{1+\left(\frac{VOC\ conc}{3.826}\right)^{4.236}}$

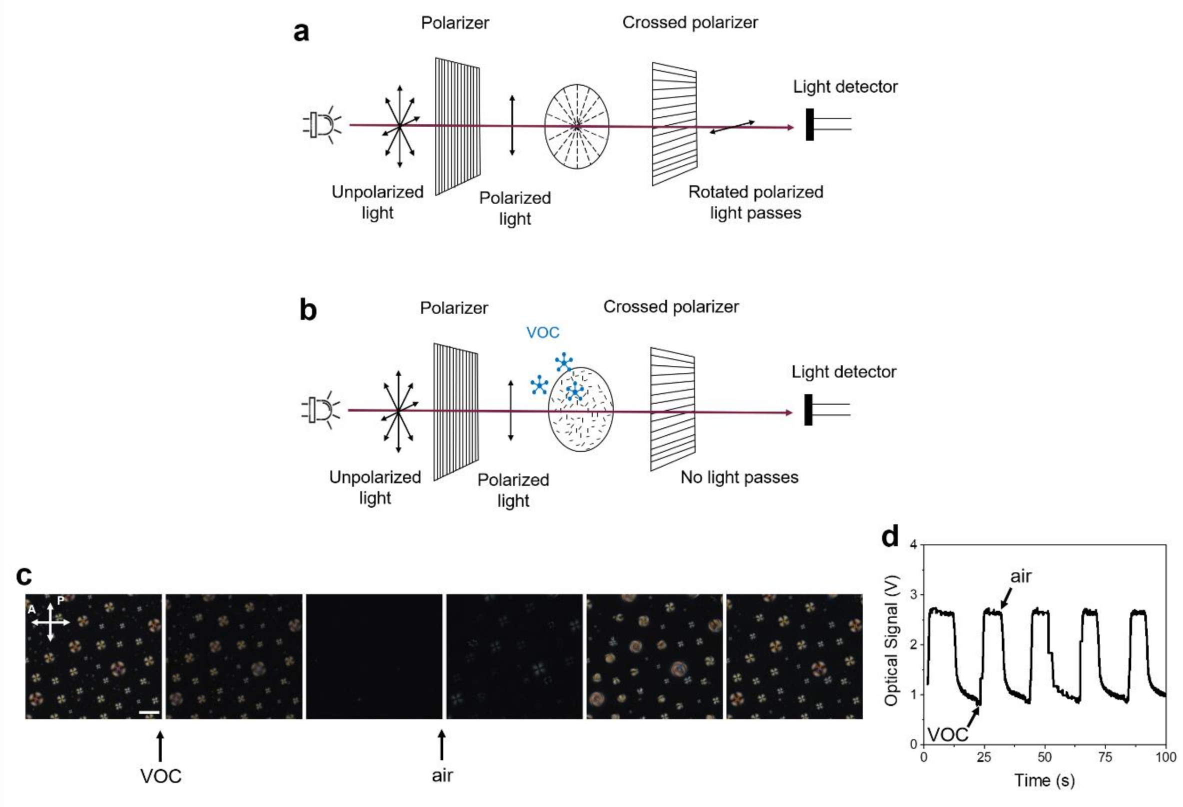

Figure S19. Optical sensing of volatile organic compounds (VOC) using radial liquid crystal (LC) droplets as probes in hybrid materials. **a)** and **b)** Representation of the optical sensing mechanism, where a hybrid film containing radial LC droplets is placed between two perpendicularly crossed polarizers and exposed alternately to air (a) and VOC (b). **c)** Polarizing optical microscopy (POM) images with crossed polarizers showing the orientational and phase transitions of the LC droplets of a hybrid film composed of gelatin, 5CB, [BMIM][DCA] and water when exposed alternately to air saturated with acetone ("VOC" arrow) and clean air ("air" arrow). Scale bar: 50 µm. **d)** Optical signal acquired from the hybrid material film in (c) with an optical signal acquisition device assembled in-house.

References

[1] A. Hussain, A.T.S.S. Semeano, S.I.C.J. Palma, A.S. Pina, J. Almeida, B.F. Medrado, A.C.C.S. Pádua, A.L. Carvalho, M. Dionísio, R.W.C. Li, H.A. Gamboa, R. V. Ulijn, J. Gruber, A.C.A. Roque, Tunable Gas Sensing Gels by Cooperative Assembly, Adv. Funct. Mater. 27 (2017) 1700803. https://doi.org/10.1002/adfm.201700803.

[2] A.T.S.S. Semeano, D.F. Maffei, S.I.C.J. Palma, R.W.C.C. Li, B.D.G.M.G.M. Franco, A.C.A.A. Roque, J. Gruber, Tilapia fish microbial spoilage monitored by a single

optical gas sensor, Food Control. 89 (2018) 72–76. https://doi.org/10.1016/j.foodcont.2018.01.025.

[3] C. Esteves, G.M.C. Santos, S.I.C.J. Palma, H.M.A. Costa, V.D. Alves, A.R. Porteira, B.M. Morais, I. Ferreira, H. Gamboa, A.C.A. Roque, Effect of film thickness in gelatin hybrid gels for artificial olfaction, Mater. Today Bio. 1 (2019) 100002. https://doi.org/10.1016/j.mtbio.2019.100002.

[4] A. Carolina Pádua, S. Palma, J. Gruber, H. Gamboa, A.C. Roque, Design and Evolution of an Opto-electronic Device for VOCs Detection, in: Proc. 11th Int. Jt. Conf. Biomed. Eng. Syst. Technol., SCITEPRESS - Science and Technology Publications, 2018: pp. 48–55. https://doi.org/10.5220/0006558100480055.

[5] R.T. da Rocha, I.G.R. Gutz, C.L. do Lago, A Low-Cost and High-Performance Conductivity Meter, J. Chem. Educ. 74 (1997) 572. https://doi.org/10.1021/ed074p572.

[6] L. Greenspan, Humidity fixed points of binary saturated aqueous solutions, J. Res. Natl. Bur. Stand. Sect. A Phys. Chem. 81A (1977) 89. https://doi.org/10.6028/jres.081A.011.

[7] Y.S. Kim, S.-C. Ha, H. Yang, Y.T. Kim, Gas sensor measurement system capable of sampling volatile organic compounds (VOCs) in wide concentration range, Sensors Actuators B Chem. 122 (2007) 211–218. https://doi.org/10.1016/J.SNB.2006.05.023.

[8] Y.-T. Lai, J.-C. Kuo, Y.-J. Yang, A novel gas sensor using polymer-dispersed liquid crystal doped with carbon nanotubes, Sensors Actuators A Phys. 215 (2014) 83–88. https://doi.org/10.1016/j.sna.2013.12.021.

[9] S.D. Hersee, J.M. Ballingall, The operation of metalorganic bubblers at reduced pressure, J. Vac. Sci. Technol. A Vacuum, Surfaces, Film. 8 (1990) 800–804. https://doi.org/10.1116/1.576921.
